# Supplementary material for: Recent hybrids recapitulate ancient hybrid outcomes
Source: Nat Commun. 2020 May 1;11:2179. doi: 10.1038/s41467-020-15641-x (PMC7195404; doi:10.1038/s41467-020-15641-x)
Supplement: Supplementary file 1 — Supplementary Inforamtion [file 41467_2020_15641_MOESM1_ESM.pdf]

Supplementary Information

Recent hybrids recapitulate ancient hybrid outcomes

Chaturvedi *et al.*

Supplementary Table 1: Locality information and sample sizes (N) for the populations included in this study. * denote new sequence data generated for this study, other sequence data were previously presented in [1].

**Locality**	**ID**	**Taxon**	**Longitude**	**Latitude**	**N**
Bonneville, UT	BST	*L. melissa*	-111.7939	41.7250	*24
Cody, WY	CDY	*L. melissa*	-108.9780	44.5121	23
Cokeville, WY	CKV	*L. melissa*	-110.9373	42.0052	10
Lander, WY	LAN	*L. melissa*	-108.3551	42.6533	24
Montague, CA	MON	*L. melissa*	-107.8162	38.3746	20
Yellow Pine, WY	YWP	*L. melissa*	-105.4006	41.2519	20
Sinclair, WY	SIN	*L. melissa*	-107.1131	41.8511	97
Victor, ID	VIC	*L. melissa*	-111.1114	43.6590	20
Bunsen Peak, WY	BNP	*L. idas*	-110.7212	44.9337	20
Garnet Peak, MT	GNP	*L. idas*	-111.2245	45.4323	98
King's Hill, MT	KHL	*L. idas*	-110.6990	46.8407	18
Soldier Creek, MT	SDC	*L. idas*	-114.6119	47.2062	20
Siyeh Creek, MT	SYV	*L. idas*	-113.6681	48.7026	20
Bald Mountain, WY	BLD	Jackson Hole	-109.7161	43.5225	*74
Bull Creek, WY	BCR	Jackson Hole	-110.5530	43.0076	46
Big Ice Cave, WY	BIC	Jackson Hole	-108.4012	45.1617	18
Blacktail Butte, WY	BTB	Jackson Hole	-110.6820	43.6382	46
Frontier Creek, WY	FRC	Jackson Hole	-109.5776	43.7206	*20
Pinnacle, WY	PIN	Jackson Hole	-109.9762	43.7401	20
Periodic Springs, WY	PSP	Jackson Hole	-110.8493	42.7468	20
Riddle Lake, WY	RDL	Jackson Hole	-110.5465	44.3617	30
Rendezvous Mtn., WY	RNV	Jackson Hole	-110.8847	43.5957	32
Dubois, WY	DBS	Hybrid zone	-109.6991	43.5623	*115

Supplementary Table 2: Summary of the observed and expected number of ancestry informative SNPs (AIMs) with the highest *L. idas* ancestry frequencies in Jackson Hole *Lycaeides* (top 10%) that were on the Z sex chromosome. Results are shown for each Jackson Hole population. We report the observed number, x-fold enrichment and (unadjusted) P value from one-sided randomization tests.

**Locality**	**No. observed**	**x-fold enrichment**	***P***
BCR	29	1.28	0.08
BIC	75	3.32	< 0.01
BLD	66	2.92	< 0.01
BTB	58	2.57	< 0.01
FRC	65	2.86	< 0.01
PIN	64	2.83	< 0.01
PSP	70	3.11	< 0.01
RDL	52	2.30	< 0.01
RNV	60	2.65	< 0.01

Supplementary Table 3: Summary of the observed and expected number of ancestry informative SNPs (AIMs) with the highest *L. melissa* ancestry frequencies in Jackson Hole *Lycaeides* (top 10%) that were on the Z sex chromosome. Results are shown for each Jackson Hole population. We report the observed number, x-fold enrichment and (unadjusted) P value from one-sided randomization tests

**Locality**	**No. observed**	**x-fold enrichment**	***P***
BCR	36	1.59	<0.01
BIC	9	0.39	0.99
BLD	8	0.35	1.00
BTB	13	0.57	0.99
FRC	6	0.26	1.00
PIN	12	0.53	0.99
PSP	12	0.52	0.98
RDL	15	0.66	0.98
RNV	15	0.65	0.99

Supplementary Table 4: Summary of the number of ancestry informative SNPs (AIMs) with the highest *L. idas* ancestry frequencies in Jackson Hole *Lycaeides* (top 10%) in or near (with 1000 bp) genes (this includes all of the sequence elements necessary to encode a functional transcript), coding sequences (including only sequences between start and stop codons; CDS), transposable elements (TEs) and proteins (matches against protein sequences). We report the observed number, x-fold enrichment and (unadjusted) P value from one-sided randomization tests.

**Category**	**No. observed**	**x-fold enrichment**	***P***
on gene	52	1.17	0.07
near gene	64	1.22	0.01
on CDS	24	1.44	0.03
near CDS	56	1.25	0.02
on TE	3	0.90	0.67
near TE	14	0.99	0.57
on protein	62	1.06	0.28
near protein	87	1.18	<0.01

Supplementary Table 5: Summary of the number of ancestry informative SNPs (AIMs) with the highest *L. melissa* ancestry frequencies in Jackson Hole *Lycaeides* (top 10%) in or near (with 1000 bp) genes (this includes all of the sequence elements necessary to encode a functional transcript), coding sequences (including only sequences between start and stop codons; CDS), transposable elements (TEs) and proteins (matches against protein sequences). We report the observed number, x-fold enrichment and (unadjusted) P value from one-sided randomization tests.

**Category**	**No. observed**	**x-fold enrichment**	$\boldsymbol{P}$
on gene	55	1.24	0.02
near gene	61	1.16	0.06
on CDS	18	1.08	0.41
near CDS	52	1.16	0.09
on TE	3	0.91	0.66
near TE	9	0.63	0.96
on protein	70	1.20	0.02
near protein	79	1.07	0.16

Supplementary Table 6: Summary of the number of ancestry informative SNPs (AIMs) with credible evidence of excess directional introgression of Jackson Hole *Lycaeides* alleles in the Dubois hybrid zone ($\alpha > 0$) in or near (with 1000 bp) genes (this includes all of the sequence elements necessary to encode a functional transcript), coding sequences (including only sequences between start and stop codons; CDS), transposable elements (TEs) and proteins (matches against protein sequences). We report the observed number, x-fold enrichment and (unadjusted) P value from one-sided randomization tests.

**Category**	**No. observed**	**x-fold enrichment**	$\boldsymbol{P}$
on gene	79	1.10	0.13
near gene	93	1.10	0.10
on cds	29	1.08	0.36
near cds	78	1.07	0.21
on TE	5	0.94	0.64
near TE	24	1.05	0.44
on protein	98	1.04	0.30
near protein	124	1.04	0.23

Supplementary Table 7: Summary of the number of ancestry informative SNPs (AIMs) with credible evidence of excess directional introgression of *L. melissa* alleles in the Dubois hybrid zone ($\alpha < 0$) in or near (with 1000 bp) genes (this includes all of the sequence elements necessary to encode a functional transcript), coding sequences (including only sequences between start and stop codons; CDS), transposable elements (TEs) and proteins (matches against protein sequences). We report the observed number, x-fold enrichment and (unadjusted) P value from one-sided randomization tests.

**Category**	**No. observed**	**x-fold enrichment**	$\boldsymbol{P}$
on gene	112	1.08	0.13
near gene	129	1.06	0.19
on CDS	53	1.36	< 0.01
near CDS	116	1.11	0.06
on TE	9	1.16	0.36
near TE	36	1.09	0.30
on protein	155	1.14	0.01
near protein	185	1.08	0.03

Supplementary Table 8: Summary of the number of ancestry informative SNPs (AIMs) with credible evidence of restricted introgression in the Dubois hybrid zone ($\beta > 0$) in or near (with 1000 bp) genes (this includes all of the sequence elements necessary to encode a functional transcript), coding sequences (including only sequences between start and stop codons; CDS), transposable elements (TEs) and proteins (matches against protein sequences). We report the observed number, x-fold enrichment and (unadjusted) P value from one-sided randomization tests.

**Category**	**No. observed**	**x-fold enrichment**	***P***
on gene	22	1.26	0.10
near gene	26	1.27	0.07
on CDS	7	1.06	0.50
near CDS	21	1.19	0.19
on TE	3	2.30	0.14
near TE	6	1.08	0.49
on protein	23	1.00	0.55
near protein	31	1.07	0.31

Supplementary Table 9: Summary of the number of ancestry informative SNPs (AIMs) with restricted introgression in the Dubois hybrid zone (10% with the highest estimates of β from `bgc`) and with high *L. idas* ancestry frequencies in Jackson Hole (top 10%) that were in or near (with 1000 bp) genes (this includes all of the sequence elements necessary to encode a functional transcript), coding sequences (including only sequences between start and stop codons; CDS), transposable elements (TEs) and proteins (matches against protein sequences). We report the observed number, x-fold enrichment and (unadjusted) P value from one-sided randomization tests.

**Category**	**No. observed**	**x-fold enrichment**	***P***
on gene	25	1.30	0.06
near gene	29	1.27	0.05
on CDS	9	1.25	0.28
near CDS	23	1.17	0.20
on TE	3	2.07	0.17
near TE	6	0.97	0.60
on protein	26	1.02	0.49
near protein	34	1.06	0.33

Supplementary Table 10: Summary of the number of ancestry informative SNPs (AIMs) with directional introgression of Jackson Hole alleles in the Dubois hybrid zone (10% with the highest estimates of α from `bgc`) and with high *L. idas* ancestry frequencies in Jackson Hole (top 10%) that were in or near (with 1000 bp) genes (this includes all of the sequence elements necessary to encode a functional transcript), coding sequences (including only sequences between start and stop codons; CDS), transposable elements (TEs) and proteins (matches against protein sequences). We report the observed number, x-fold enrichment and (unadjusted) P value from one-sided randomization tests.

**Category**	**No. observed**	**x-fold enrichment**	***P***
on gene	9	1.08	0.46
near gene	12	1.22	0.24
on CDS	5	1.59	0.20
near CDS	11	1.30	0.18
on TE	0	0.00	1.00
near TE	2	0.75	0.77
on protein	12	1.10	0.41
near protein	16	1.16	0.23

Supplementary Table 11: Summary of the number of ancestry informative SNPs (AIMs) with directional introgression of Jackson Hole alleles in the Dubois hybrid zone (10% with the highest estimates of α from `bgc`) and with high *L. melissa* ancestry frequencies in Jackson Hole (top 10%) that were in or near (with 1000 bp) genes (this includes all of the sequence elements necessary to encode a functional transcript), coding sequences (including only sequences between start and stop codons; CDS), transposable elements (TEs) and proteins (matches against protein sequences). We report the observed number, x-fold enrichment and (unadjusted) P value from one-sided randomization tests.

**Category**	**No. observed**	**x-fold enrichment**	***P***
on gene	14	1.38	0.09
near gene	15	1.24	0.17
on CDS	6	1.56	0.18
near CDS	15	1.45	0.05
on TE	1	1.30	0.55
near TE	2	0.61	0.86
on protein	15	1.12	0.34
near protein	17	1.00	0.59

Supplementary Table 12: Summary of protein descriptions from `interproscan` for ancestry informative SNPs (AIMs) with high *L. idas* ancestry in the ancient, Jackson Hole hybrids and restricted introgression in the contemporary Dubois hybrid zone (top 10% in each case). For each gene, the linkage group (LG) (LG 23 = Z), genome scaffold, position(s) of SNP(s) in or near (within 1000 bp) the gene (SNPs) and start and stop of the gene annotation are given along with the IPR number and associated description from `interproscan`.

LG	Scaffold	SNPs	Start	Stop	IPR #	Description
1	1628	11727258	11720849	11726566	IPR007231	Nucleoporin interacting component Nup93/Nic96
1	1628	12893859	12894703	12894888	NA	NA
2	11	8117866	8115532	8118472	IPR004117	Olfactory receptor, insect
3	1646	12073166,	12053895	12075705	IPR011989	Armadillo-like helical
		12073190			IPR026818	Adenomatous polyposis coli (APC) family
					IPR011989	Armadillo-like helical
					IPR026818	Adenomatous polyposis coli (APC) family
7	1642	9017721	9012690	9023798	IPR001781	Zinc finger, LIM-type
9	1641	9383455	9371915	9383193	IPR004878	Otopetrin
10	1639	8438185	8435143	8440922	IPR023102	Fatty acid synthase, domain 2
10	1639	10026163	10023889	10032998	IPR000648	Oxysterol-binding protein
					IPR011993	PH-like domain superfamily
10	1639	12748595	12739317	12751985	IPR036236	Zinc finger C2H2 superfamily
11	4	12406546	12403487	12420293	IPR036388	Winged helix-like DNA-binding domain superfamily
					IPR037241	E2F-DP heterodimerization region
11	4	12406549			IPR037241	E2F-DP heterodimerization region
					IPR037241	E2F-DP heterodimerization region
12	833	6668579	6667703	6667757	NA	NA
23	1631	1090637	1091079	1091110	NA	NA
23	1631	4238467	4237518	4238564	IPR011011	Zinc finger, FYVE/PHD-type
23	1631	5365561	5365704	5366438	NA	NA
23	1631	5516528	5488340	5561251	IPR027417	P-loop containing nucleoside triphosphate hydrolase
23	1631	6146721	6146944	6146977	NA	NA
23	1631	7534776	7528709	7535929	NA	NA
23	1631	7688497	7684329	7697467	IPR011989	Armadillo-like helical
					IPR027651	FH1/FH2 domain-containing protein 3
23	1631	8161313	8155537	8174517	IPR036865	CRAL-TRIO lipid binding domain superfamily
					IPR008936	Rho GTPase activation protein
23	1631	9616373	9611236	9626847	IPR036179	Immunoglobulin-like domain superfamily
23	1631	10080466,	10057941	10097025	IPR036179	Immunoglobulin-like domain superfamily
		10096614,				
		10096721				
23	1631	12342151	12341301	12341547	NA	NA
23	1631	12682671	12681749	12681828	NA	NA
23	1631	13728571	13729333	13729356	NA	NA
23	1631	13908210	13908744	13908776	NA	NA
23	1631	13944328	13943280	13943458	NA	NA
23	1631	14175992	14174137	14174993	NA	NA
		14799474			NA	NA
23	1631	14992849	14980060	15000961	IPR013320	Concanavalin A-like lectin/glucanase domain superfamily
23	1631	15003188	15002491	15002528	NA	NA
23	1631	15044549,	15044399	15057269	NA	NA
		15347619			IPR002017	Spectrin repeat
					IPR011993	PH-like domain superfamily
					IPR035899	Dbl homology (DH) domain superfamily
23	1631	15428089,	15418766	15469764	NA	NA
		15428102				
23	1631	15630833	15628588	15650639	IPR008927	6-phosphogluconate dehydrogenase-like, C-terminal domain superfamily
					IPR036291	NAD(P)-binding domain superfamily
23	1631	16563238,	16562585	16571768	NA	NA
		16563245				
23	1631	16742878	16742257	16742291	NA	NA
23	1631	17005201	16989784	17005380	NA	NA
23	1631	17266869	17267147	17267186	NA	NA
23	1631	18504663	18503673	18503704	NA	NA
23	1631	20910914	20911725	20925281	IPR039170	5'-AMP-activated protein kinase subunit gamma-2
23	1631	21035262,	21034582	21034639	NA	NA
		21035340				
23	1631	21250737,	21250378	21250415	NA	NA
		21250758				
23	1631	21303464	21296234	21303124	IPR011009	Protein kinase-like domain superfamily

Supplementary Table 13: Summary of gene ontology (GO) terms (i.e., classifications) for ancestry informative SNPs (AIMs) with high *L. idas* ancestry in the ancient, Jackson Hole hybrids and restricted introgression in the contemporary Dubois hybrid zone (top 10% in each case). For each gene, the linkage group (LG), genome scaffold, position(s) of SNP(s) in or near (within 1000 bp) the gene (SNPs) and start and stop of the gene annotation are given along with the GO term numbers (GO #) and descriptions. Here, results are shown for the 22 autosomes. Symbols denote whether each term corresponds to a biological process (-b), molecular function (-m), or cellular component (-c).

**LG**	**Scaffold**	**SNPs**	**Start**	**Stop**	**GO #**	**Description**
1	1628	11727258	11720849	11726566	GO:0005643-c	Nuclear pore
					GO:0017056-m	Structural constituent of nuclear pore
1	1628	12893859	12894703	12894888	NA	NA
2	11	8117866	8115532	8118472	GO:0004984-m	Olfactory receptor activity
					GO:0005549-m	Odorant binding
					GO:0007608-b	Sensory perception of smell
					GO:0016020-c	Membrane
3	1646	12073166,	12053895	12075705	GO:0005515-m	Protein binding
		12073190			GO:0008013-m	Beta-catenin binding
					GO:0016055-b	Wnt signaling pathway
					GO:0030178-b	Negative regulation of Wnt signaling pathway
7	1642	9017721	9012690	9023798	NA	NA
9	1641	9383455	9371915	9383193	NA	NA
10	1639	8438185	8435143	8440922	GO:0004312-m	Fatty acid synthase activity
					GO:0009058-b	Biosynthetic process
					GO:0016788-m	Hydrolase activity, acting on ester bonds
10	1639	10026163	10023889	10032998	NA	NA
10	1639	12748595	12739317	12751985	GO:0003676-m	Nucleic acid binding
11	4	12406546	12403487	12420293	GO:0003700-m	DNA-binding transcription factor activity
					GO:0005667-c	Transcription factor complex
					GO:0006355-b	Regulation of transcription, DNA-templated
					GO:0046983-m	Protein dimerization activity
11	4	12406549	12403487	12420293	GO:0003700-m	DNA-binding transcription factor activity
					GO:0005667-c	Transcription factor complex
					GO:0006355-b	Regulation of transcription, DNA-templated
					GO:0046983-m	Protein dimerization activity
12	833	6668579	6667703	6667757	NA	NA

Supplementary Table 14: Summary of gene ontology (GO) terms (i.e., classifications) for ancestry informative SNPs (AIMs) which had high *L. idas* ancestry in the ancient, Jackson Hole hybrids and restricted introgression in the contemporary Dubois hybrid zone (top 10% in each case). For each gene, the linkage group (LG) (LG 23 = Z), genome scaffold, position(s) of SNP(s) in or near (within 1000 bp) the gene (SNPs) and start and stop of the gene annotation are given along with the GO term numbers (GO #) and descriptions. Here, results are shown for the Z chromosome (LG = 23). Symbols denote whether each term corresponds to a biological process (-b), molecular function (-m), or cellular component (-c).

**LG**	**Scaffold**	**SNPs**	**Start**	**Stop**	**GO #**	**Description**
23	1631	1090637	1091079	1091110	NA	NA
23	1631	4238467	4237518	4238564	NA	NA
23	1631	5365561	5365704	5366438	NA	NA
23	1631	5516528	5488340	5561251	GO:0003777-m	Microtubule motor activity
					GO:0005524-m	ATP binding
					GO:0005858-c	Axonemal dynein complex
					GO:0007018-b	Microtubule-based movement
					GO:0016887-m	ATPase activity
					GO:0060285-b	Cilium-dependent cell motility
23	1631	6146721	6146944	6146977	NA	NA
23	1631	7534776	7528709	7535929	NA	NA
23	1631	7688497	7684329	7697467	GO:0007015-b	Actin filament organization
23	1631	8161313	8155537	8174517	GO:0007165-b	Signal transduction
23	1631	9616373	9611236	9626847	NA	NA
23	1631	10080466, 10096614, 10096721	10057941	10097025	NA	NA
23	1631	12342151	12341301	12341547	NA	NA
23	1631	12682671	12681749	12681828	NA	NA
23	1631	13728571	13729333	13729356	NA	NA
23	1631	13908210	13908744	13908776	NA	NA
23	1631	13944328	13943280	13943458	NA	NA
23	1631	14175992	14174137	14174993	NA	NA
23	1631	14799474	14799137	14799168	NA	NA
23	1631	14992849	14980060	15000961	NA	NA
23	1631	15003188	15002491	15002528	NA	NA
23	1631	15044549	15044399	15057269	NA	NA
23	1631	15347619	15326999	15377180	GO:0005089-m	Rho guanyl-nucleotide exchange factor activity
					GO:0005515-m	Protein binding
					GO:0035023-b	Regulation of Rho protein signal transduction
23	1631	15428089, 15428102	15418766	15469764	NA	NA
23	1631	15630833	15628588	15650639	GO:0004616-m	Phosphogluconate dehydrogenase (decarboxylating) activity
					GO:0006098-b	Pentose-phosphate shunt
					GO:0016491-m	Oxidoreductase activity
					GO:0050661-m	NADP binding
					GO:0055114-b	Oxidation-reduction process
23	1631	16563238, 16563245	16562585	16571768	NA	NA
23	1631	16742878	16742257	16742291	NA	NA
23	1631	17005201	16989784	17005380	NA	NA
23	1631	17266869	17267147	17267186	NA	NA
23	1631	18504663	18503673	18503704	NA	NA
23	1631	20910914	20911725	20925281	GO:0032559-m	Regulation of protein serine/threonine kinase activity
23	1631	21035262 21035340	21034582	21034639	NA	NA
23	1631	21250737 21250758	21250378	21250415	NA	NA
23	1631	21303464	21296234	21303124	GO:0004672-m	Protein kinase activity
					GO:0005524-m	ATP binding
					GO:0006468-b	Protein phosphorylation

Supplementary Table 15: Summary of protein descriptions from `interproscan` for ancestry informative SNPs (AIMs) with high *L. idas* ancestry in the ancient, Jackson Hole hybrids and directional Jackson Hole *Lycaeides* introgression ($\alpha > 0$) in the contemporary Dubois hybrid zone (top 10% in each case). For each gene, the linkage group (LG) (LG 23 = Z), genome scaffold, position(s) of SNP(s) in or near (within 1000 bp) the gene (SNPs) and start and stop of the gene annotation are given along with the IPR number and associated description from `interproscan`.

LG	Scaffold	SNPs	Start	Stop	IPR #	Description
2	11	6234637	6215867	6240330	IPR001930	Peptidase M1, alanine aminopeptidase/leukotriene A4 hydrolase
					IPR024571	ERAP1-like C-terminal domain
2	11	15712950	15710210	15713015	IPR009851	Modifier of rudimentary, Modr
2	11	15712950	NA	NA	IPR037859	Vacuolar protein sorting-associated protein 37
4	1648	13426790	13426596	13426775	NA	NA
7	1642	9023853	9012690	9025104	IPR001781	Zinc finger, LIM-type
9	1641	9384153	9371915	9383193	IPR004878	Otopetrin
10	1639	8378132	8374436	8380586	NA	NA
10	1639	13668785	13668901	13668943	NA	NA
12	833	6668579	6667703	6667757	NA	NA
13	1632	6146628	6145294	6150912	IPR011047	Quinoprotein alcohol dehydrogenase-like superfamily
16	503	6014039	NA	NA	NA	NA
23	1631	1200324	1197667	1204058	IPR036719	Neurotransmitter-gated ion-channel transmembrane domain superfamily
23	1631	1200324	NA	NA	IPR036734	Neurotransmitter-gated ion-channel ligand-binding domain superfamily
23	1631	2570345	2570129	2570178	NA	NA
23	1631	4238526	4237518	4238564	IPR011011	Zinc finger, FYVE/PHD-type
23	1631	9958857	9955713	9968188	IPR027266	GTP-binding protein TrmE/Glycine cleavage system T protein, domain 1
23	1631	9958857	NA	NA	IPR036188	FAD/NAD(P)-binding domain superfamily
23	1631	10080421	10057941	10097025	IPR036179	Immunoglobulin-like domain superfamily
23	1631	10080466	10057941	10097025	IPR036179	Immunoglobulin-like domain superfamily
23	1631	12954191	12954213	12954434	NA	NA
23	1631	14218357	14216920	14217629	NA	NA
23	1631	14760795	14746596	14779181	IPR000034	Laminin IV
					IPR002049	Laminin EGF domain
					IPR036055	LDL receptor-like superfamily
					IPR036179	Immunoglobulin-like domain superfamily
23	1631	15408704	15409126	15409167	NA	NA
23	1631	15428179	15418766	15469764	NA	NA
23	1631	20910914	20911725	20925281	IPR000644	CBS domain
					IPR039170	5'-AMP-activated protein kinase subunit gamma-2

Supplementary Table 16: Summary of gene ontology (GO) terms (i.e., classifications) for ancestry informative SNPs (AIMs) with high *L. idas* ancestry in the ancient, Jackson Hole hybrids and directional Jackson Hole *Lycaeides* introgression ($\alpha > 0$) in the contemporary Dubois hybrid zone (top 10% in each case). For each gene, the linkage group (LG) (LG 23 = Z), genome scaffold, position(s) of SNP(s) in or near (within 1000 bp) the gene (SNPs) and start and stop of the gene annotation are given along with the GO term numbers (GO #) and descriptions. Symbols denote whether each term corresponds to a biological process (-b), molecular function (-m), or cellular component (-c).

**LG**	**Scaffold**	**SNPs**	**Start**	**Stop**	**GO #**	**Description**
2	11	6234637	6234637	6215867	GO:0006508-b	Proteolysis
					GO:0008237-m	Metallopeptidase activity
					GO:0008270-m	Zinc ion binding
2	11	15712950	15710210	15713015	GO:0000813-c	ESCRT I complex
					GO:0032509-b	Endosome transport via multivesicular body sorting pathway
4	1648	13426790	13426790	13426596	NA	NA
7	1642	9023853	9023853	9012690	NA	NA
9	1641	9384153	9384153	9371915	NA	NA
10	1639	8378132	8374436	8380586	NA	NA
10	1639	13668785	13668785	13668901	NA	NA
12	833	6668579	6667703	6667757	NA	NA
13	1632	6146628	6146628	6145294	NA	NA
16	503	6014039	NA	NA	NA	NA
23	1631	1200324	1197667	1204058	GO:0004888-m	Transmembrane signaling receptor activity
					GO:0004890-m	GABA-A receptor activity
					GO:0005216-m	Ion channel activity
					GO:0005230-m	Extracellular ligand-gated ion channel activity
					GO:0006811-b	Ion transport
					GO:0016020-c	Membrane
					GO:0016021-c	Integral component of membrane
					GO:0034220-b	Ion transmembrane transport
23	1631	2570345	2570129	2570178	NA	NA
23	1631	4238526	4237518	4238564	NA	NA
23	1631	9958857	9955713	9968188	GO:0005515-m	Protein binding
					GO:0016491-m	Oxidoreductase activity
					GO:0055114-b	Oxidation-reduction process
23	1631	10080421,<br>10080466	10057941	10097025	NA	NA
23	1631	12954191	12954213	12954434	NA	NA
23	1631	14218357	14216920	14217629	NA	NA
23	1631	14760795	14746596	14779181	GO:0005515-m	Protein binding
23	1631	15408704	15409126	15409167	NA	NA
23	1631	15428179	15418766	15469764	NA	NA
23	1631	20910914	20911725	20925281	GO:0032559-m	Adenyl ribonucleotide binding
					GO:0071900-b	Regulation of protein serine/threonine kinase activity

Supplementary Table 17: Summary of protein descriptions from `interproscan` for ancestry informative SNPs (AIMs) with high *L. melissa* ancestry in the ancient, Jackson Hole hybrids and directional Jackson Hole *Lycaeides* introgression ($\alpha > 0$) in the contemporary Dubois hybrid zone (top 10% in each case). For each gene, the linkage group (LG) (LG 23 = Z), genome scaffold, position(s) of SNP(s) in or near (within 1000 bp) the gene (SNPs) and start and stop of the gene annotation are given along with the IPR number and associated description from `interproscan`.

LG	Scaffold	SNPs	Start	Stop	IPR #	Description
1	1628	2792953	2782773	2797741	IPR004274	FCP1 homology domain
1	1628	13677379	NA	NA	NA	NA
2	11	7335097	7335985	7336153	NA	NA
2	11	8691829	8691651	8691894	NA	NA
2	11	10860558,	10855256	10861383	IPR013083	Zinc finger
		10860564			IPR034732	RING/FYVE/PHD-type
4	1648	447128	447024	447052	NA	NA
7	1642	11407787	11392125	11409041	IPR011701	Major facilitator superfamily
8	1645	9366622	9365131	9365852	NA	NA
8	1645	9587985	9587772	9588024	NA	NA
8	1645	15122056	NA	NA	NA	NA
8	1645	15122098	NA	NA	NA	NA
9	1641	2835785	2833944	2841076	IPR009033	Calreticulin/calnexin, P domain superfamily
9	1641	13685914	13678341	13685134	IPR006621	Nose resistant-to-fluoxetine protein, N-terminal
10	1639	12335336	12333398	12336053	IPR029526	PiggyBac transposable element-derived protein
11	4	4190884	4190808	4190860	NA	NA
17	309	8389080,	8389311	8389348	NA	NA
		8389096			NA	NA
17	309	12340022	12331408	12343477	IPR004210	BESS motif
					IPR006578	MADF domain
	758	2023	NA	NA	NA	NA
18	1647	3319221,	3316087	3324488	IPR011032	GroES-like superfamily
		3319229,			IPR013154	Alcohol dehydrogenase, N-terminal
		3319267			IPR020843	Polyketide synthase, enoylreductase domain
					IPR036292	NAD(P)-binding domain superfamily
21	1095	7019482,	7019198	7025723	IPR039767	RalA-binding protein 1
		7019493,				
		7019553				
23	1631	6116900	6109396	6117970	IPR000159	Ras-associating (RA) domain,Pleckstrin homology domain

Supplementary Table 18: Summary of gene ontology (GO) terms (i.e., classifications) for ancestry informative SNPs (AIMs) with high *L. melissa* ancestry in the ancient, Jackson Hole hybrids and directional Jackson Hole *Lycaeides* introgression ($\alpha > 0$) in the contemporary Dubois hybrid zone (top 10% in each case). For each gene, the linkage group (LG) (LG 23 = Z), genome scaffold, position(s) of SNP(s) in or near (within 1000 bp) the gene (SNPs) and start and stop of the gene annotation are given along with the GO term numbers (GO #) and descriptions. Symbols denote whether each term corresponds to a biological process (-b), molecular function (-m), or cellular component (-c).

**LG**	**Scaffold**	**SNPs**	**Start**	**Stop**	**GO #**	**Description**
1	1628	2792953	2782773	2797741	GO:0016791-m	Phosphatase activity
1	1628	13677379	NA	NA	NA	NA
2	11	7335097	7335985	7336153	NA	NA
2	11	8691829	8691651	8691894	NA	NA
2	11	10860558,	10855256	10861383	NA	NA
		10860564			NA	NA
4	1648	447128	447024	447052	NA	NA
7	1642	11407787	11392125	11409041	GO:0016021-c	Integral component of membrane
8	1645	9366622	9365131	9365852	NA	NA
8	1645	9587985	9587772	9588024	NA	NA
8	1645	15122056	NA	NA	NA	NA
8	1645	15122098	NA	NA	NA	NA
9	1641	2835785	2833944	2841076	GO:0005509	calcium ion binding
					GO:0051082	protein folding
9	1641	13685914	13678341	13685134	NA	NA
10	1639	12335336	12333398	12336053	NA	NA
11	4	4190884	4190808	4190860	NA	NA
17	309	8389080,	8389311	8389348	NA	NA
		8389096			NA	NA
17	309	12340022	12331408	12343477	GO:0003677-m	DNA binding
	758	2023	NA	NA	NA	NA
18	1647	3319221,	3316087	3324488	GO:0016491-m	Oxidoreductase activity
		3319229,				
		3319267				
21	1095	7019482,	7019198	7025723	GO:0005096-m	GTPase activator activity
		7019493,				
		7019553				
23	1631	6116900	6109396	6117970	GO:0007165-b	Signal transduction

Supplementary Table 19: Table gives a list of sequences used from `LepBase` version 4 to create the protein homology file for genome annotation using `maker` pipeline.

	**Sequence name**
1	Amyelois_transitella_v1_-_proteins.fa
2	Bicyclus_anynana_nBa.0.1_-_proteins.fa
3	Bicyclus_anynana_v1.2_-_proteins.fa
4	Bombyx_mori_ASM15162v1_-_proteins.fa
5	Calycopis_cecrops_v1.1_-_proteins.fa
6	Chilo_suppressalis_CsuOGS1.0_-_proteins.fa
7	Danaus_plexippus_v3_-_proteins.fa
8	Heliconius_erato_demophoon_v1_-_proteins.fa
9	Heliconius_erato_lativitta_v1_-_proteins.fa
10	Heliconius_melpomene_melpomene_Hmel1_-_proteins.fa
11	Heliconius_melpomene_-_proteins.fa
12	Junonia_coenia_JC_v1.0_-_proteins.fa
13	Lerema_accius_v1.1_-_proteins.fa
14	Limnephilus_lunatus_v1_-_proteins.fa
15	Manduca_sexta_Msex_1.0_-_proteins.fa
16	Operophtera_brumata_v1_-_proteins.fa
17	Papilio_glaucus_v1.1_-_proteins.fa
18	Papilio_machaon_Pap_ma_1.0_-_proteins.fa
19	Papilio_polytes_Ppol_1.0_-_proteins.fa
20	Papilio_polytes_Ppol_1.0_Refseq_-_proteins.fa
21	Papilio_xuthus_Pap_xu_1.0_-_proteins.fa
22	Papilio_xuthus_Pxut_1.0_-_proteins.fa
23	Papilio_xuthus_Pxut_1.0_Refseq_-_proteins.fa
24	Phoebis_sennae_v1.1_-_proteins.fa
25	Plodia_interpunctella_v1_-_proteins.fa
26	Plutella_xylostella_DBM_FJ_v1.1_-_proteins.fa
27	Plutella_xylostella_pacbiov1_-_proteins.fa

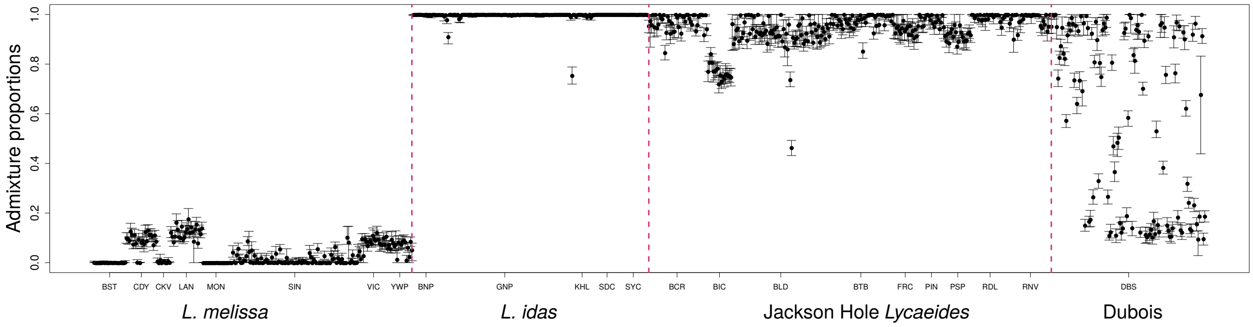

Supplementary Figure 1: Admixture proportion estimates from `entropy` with $k = 2$ source populations (N = 835 butterflies from 23 populations). Points (posterior median) and error bars (95% credible intervals) denote the estimates of the proportion of the genome with *L. idas* ancestry. Tick marks below the plots identify populations based on the population abbreviations in Supplementary Table 1. Individuals with low coverage are not shown in this figure.

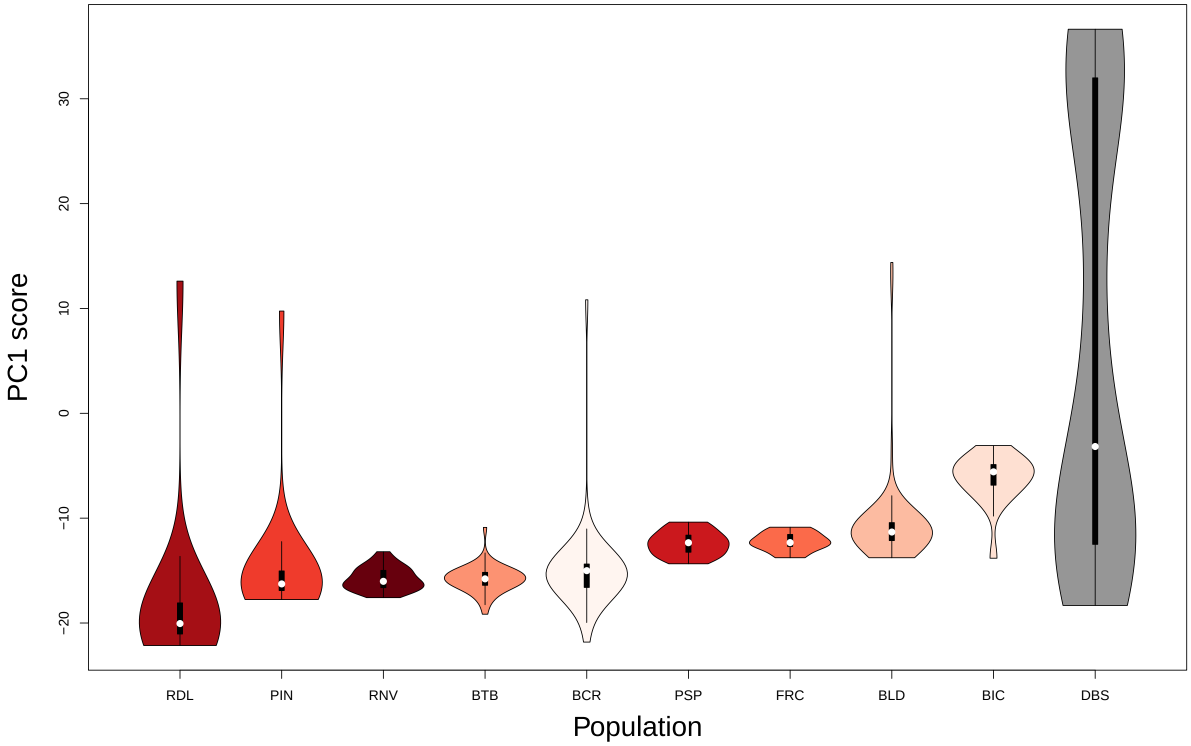

Supplementary Figure 2: Violin plots show the distribution of genetic PC1 scores among individuals for each population ($N = 306$ Jackson Hole *Lycaeides* from nine populations and 115 butterflies from Dubois). Here, white dots denote the median, black boxes denote the 1st and 3rd quartile, vertical bars extend to the minimum and maximum value or 1.5× the interquartile range, and kernel densities show the full data distribution. Tick marks below the plots identify populations based on the population abbreviations in Supplementary Table 1.

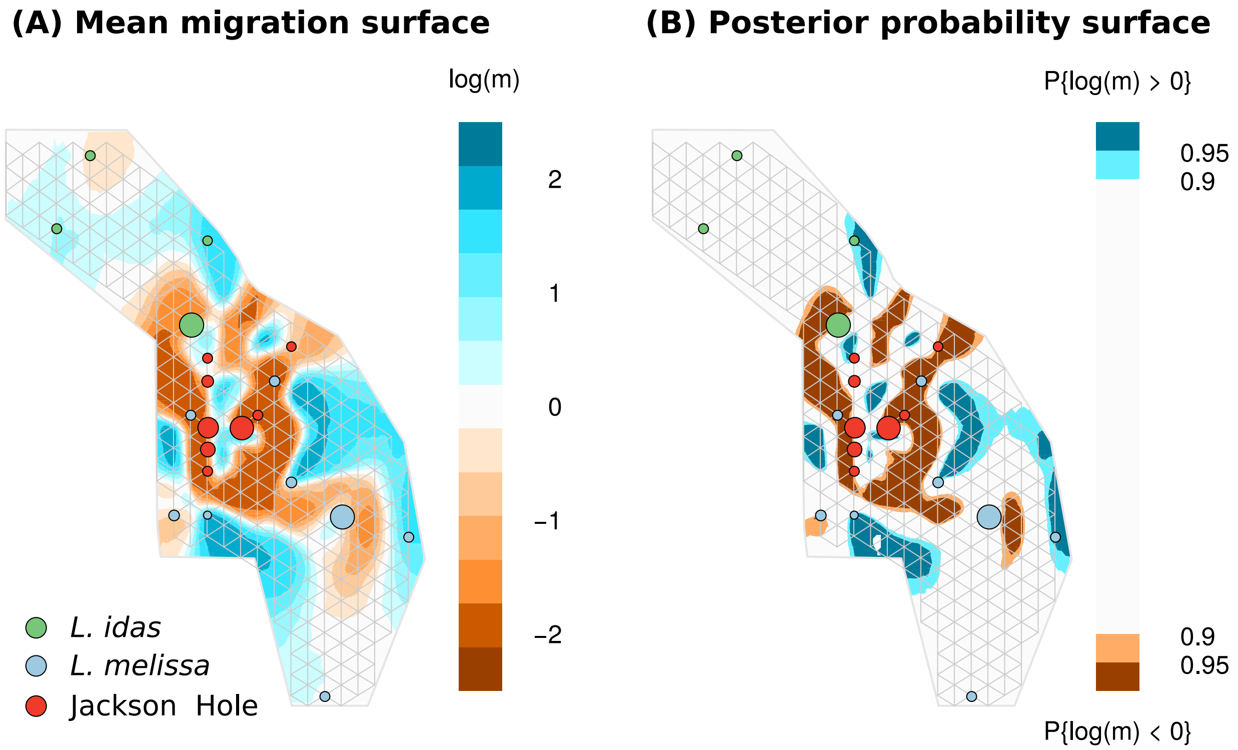

Supplementary Figure 3: Plots summarize effective migration rates among *L. idas*, *L. melissa* and Jackson hole populations in terms of (A) the posterior mean effective migration point estimate and (B) the posterior probability that the log effective migration rate (m) is greater than or less than 0. The grid denotes 300 hypothetical demes (nodes) potentially connected by migration (edges). Point sizes are proportional to sample sizes, and individual points (demes) sometimes included individuals from two nearby populations. Dark brown regions of the map indicate reduced effective migration rates relative to expectations if gene flow were constant per unit distance across the map. This shows a barrier to gene flow centered on the Jackson Hole populations (but not for paths between different Jackson Hole populations within this region), and thus differs from a simple isolation-by-distance pattern. Dubois was not included in this analysis.

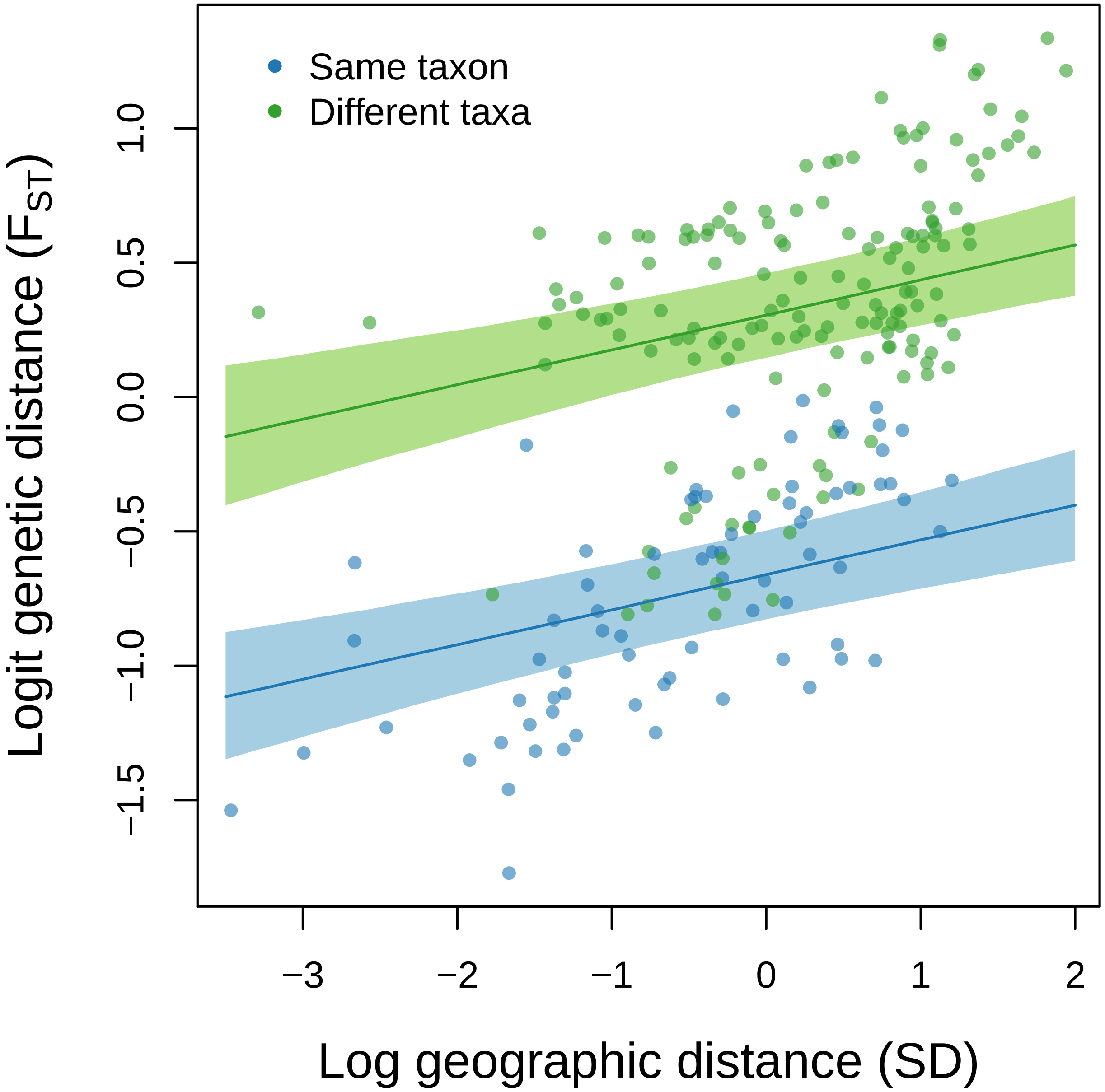

Supplementary Figure 4: Plot shows the relationship between geographic and genetic distances among populations. We show centered logit F_{ST} as a function of standardized (centered and SD = 1) log geographic distance. Points are colored to denote whether each of the population pairs ($N = \frac{22\times21}{2} = 231$ pairs excluding Dubois) are of the same or different nominal taxa (*L. melissa*, *L. idas* or Jackson Hole). Lines and shaded areas denote Bayesian point estimates (posterior median) and 90% credible intervals for the relationship between geographic and genetic distance. A model including geographic distance and same vs. different taxon was preferred (DIC = 92.82) relative to one with only taxon (DIC = 98.67) or only geographic distance (DIC = 360.9). Thus, whereas there is a pattern of isolation-by-distance within taxa, genetic distances between heterospecific taxa (including Jackson Hole vs. *L. idas* or *L. melissa*) are higher than expected from geographic distances alone. Dubois was not included in this analysis.

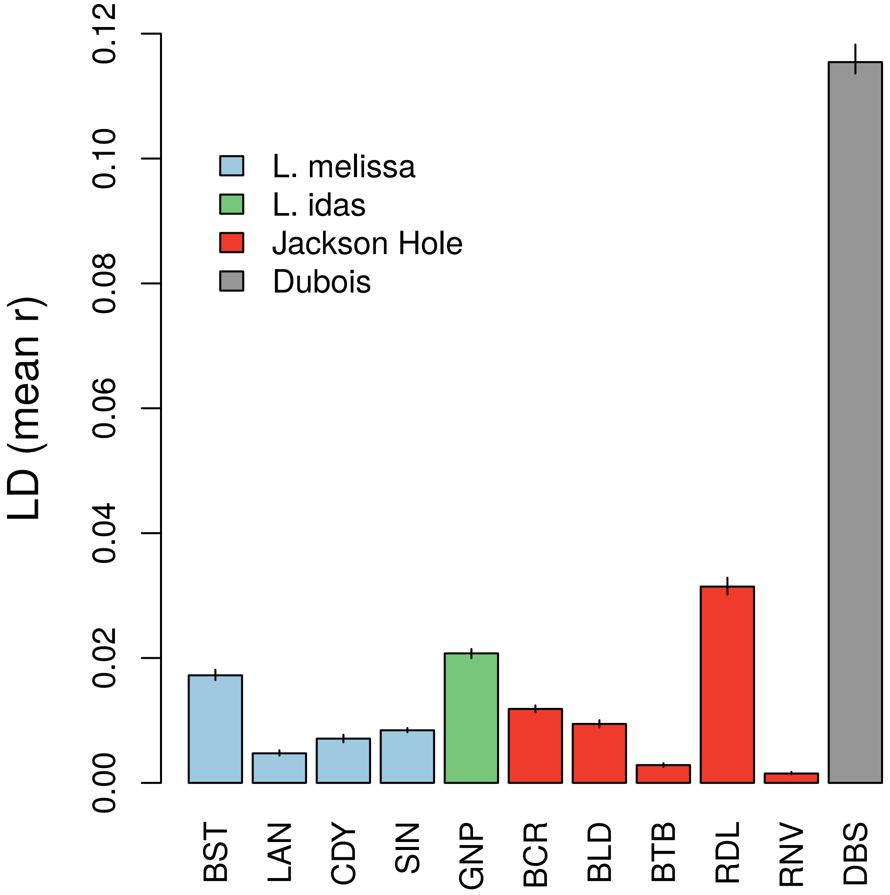

Supplementary Figure 5: Bars denote the mean value of linkage disequilibrium (LD) for all pairs of ancestry informative SNPs (AIMs) for each population (676,866 AIM pairs), vertical lines give ± standard errors. Here, LD was measured as the Pearson correlation between genotypes at pairs of loci (regardless of physical linkage). LD was calculated based on posterior estimates of genotypes from `entropy`. Genotypes were first polarized such that positive LD indicates a positive association between pairs of alleles that were more common in each of the parental species (i.e., coupling LD). We report r rather than r^2 to retain this sign information. AIMs were defined as those SNPs with an allele frequency difference of at least 0.3 between *L. idas* and *L. melissa*. Only populations with sample sizes of at least 20 were included in this analysis (N = 609 butterflies from 11 populations). Population abbreviations are defined in Supplementary Table 1.

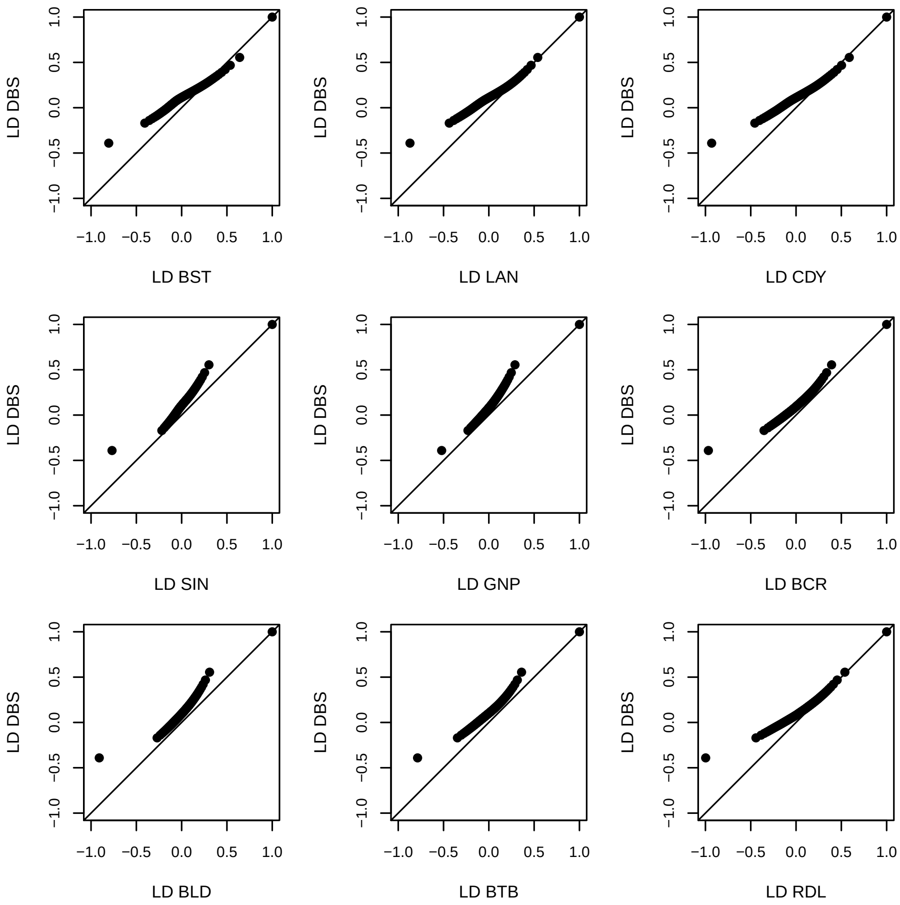

Supplementary Figure 6: Quantile-quantile plots show the distribution of linkage disequilibrium (LD) for all pairs of ancestry informative SNPs (AIMs) for each population when compared to Dubois. Here, LD was measured as the Pearson correlation between genotypes at pairs of loci (regardless of physical linkage). LD was calculated based on posterior estimates of genotypes from `entropy`. Genotypes were first polarized such that positive LD indicates a positive association between pairs of alleles that were more common in each of the parental species (i.e., coupling LD). We report r rather than r^2 to retain this sign information. AIMs were defined as those SNPs with an allele frequency difference of at least 0.3 between *L. idas* and *L. melissa*. Only populations with sample sizes of at least 20 were included in this analysis. Population abbreviations and sample sizes are given in Supplementary Table 1.

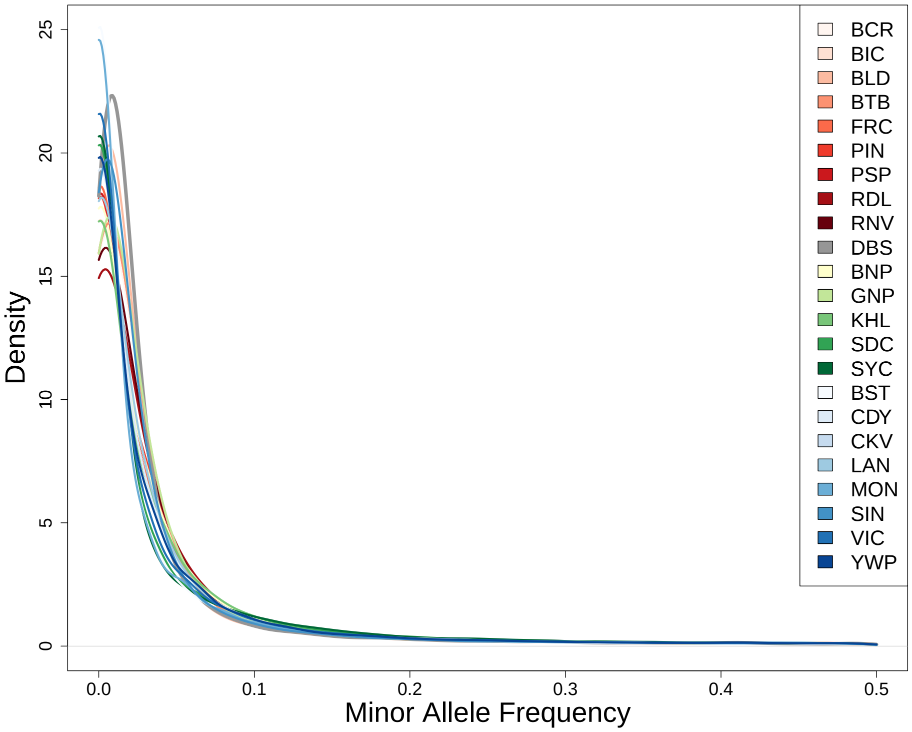

Supplementary Figure 7: Density plot shows the estimated minor allele frequency distribution across all loci (N = 39,139 SNPs) for all 23 populations included in this study. The population abbreviations are defined in Supplementary Table 1. Mean minor allele frequencies for the subset of ancestry informative SNPs (AIMs) are show in in Supplementary Figure 8.

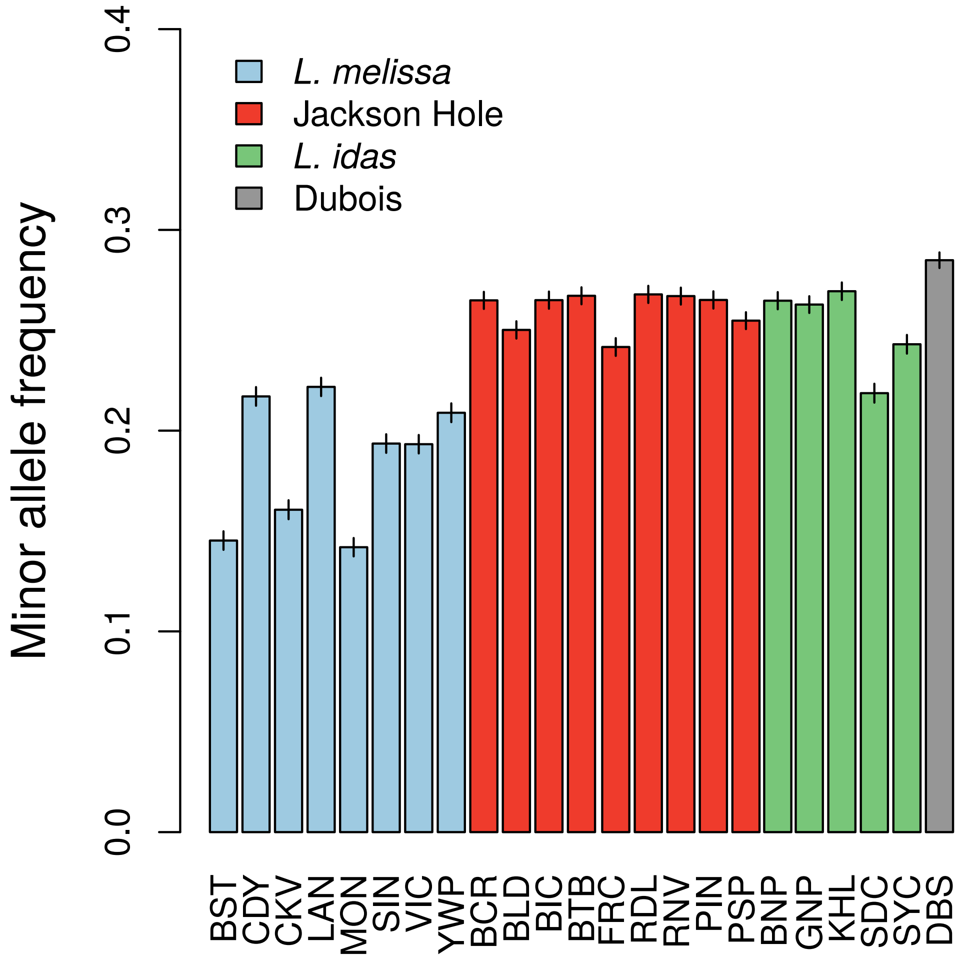

Supplementary Figure 8: Bars show the mean estimated minor allele frequency distribution for ancestry informative SNPs (AIMs) (N = 1164) for all 23 populations included in this study. Vertical lines give ± standard errors for the means. The full distribution of minor allele frequencies is depicted in Supplementary Figure 7. Population abbreviations are defined in Supplementary Table 1.

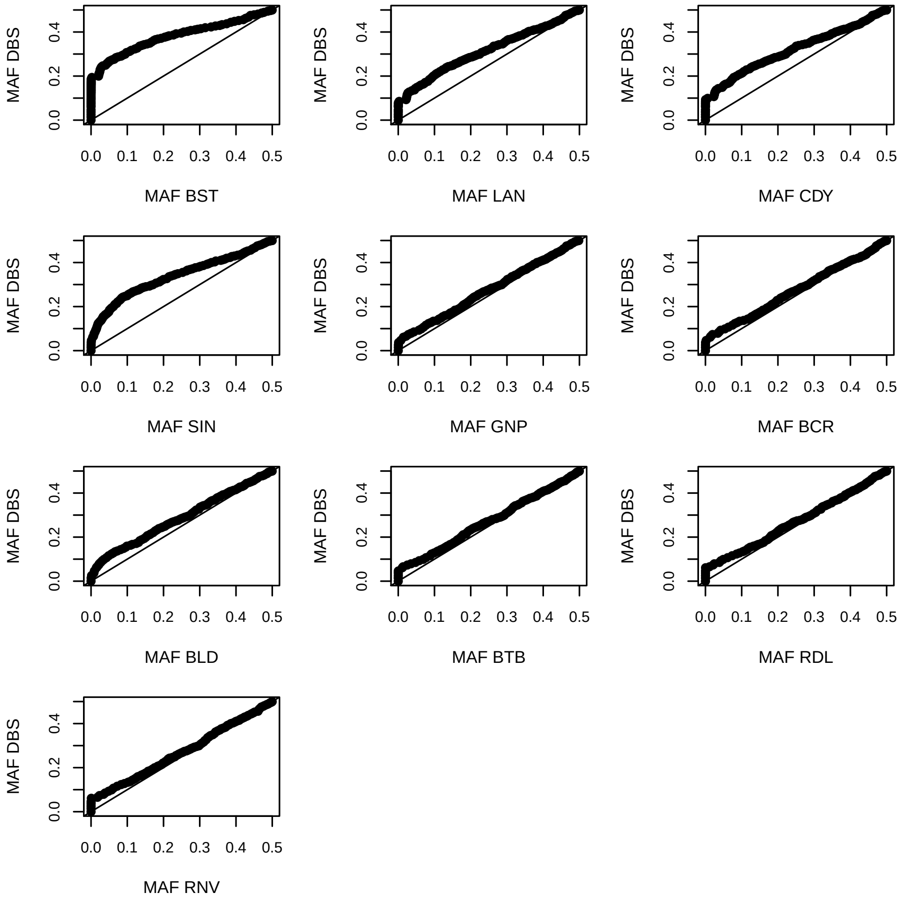

Supplementary Figure 9: Quantile-quantile plots show the distribution of minor allele frequency (MAF) for all pairs of ancestry informative SNPs (AIMs) for each population when compared to Dubois. Here, AIMs were defined as those SNPs with an allele frequency difference of at least 0.3 between *L. idas* and *L. melissa*. Only populations with sample sizes of at least 20 were included in this analysis. Population abbreviations and sample sizes are given in Supplementary Table 1.

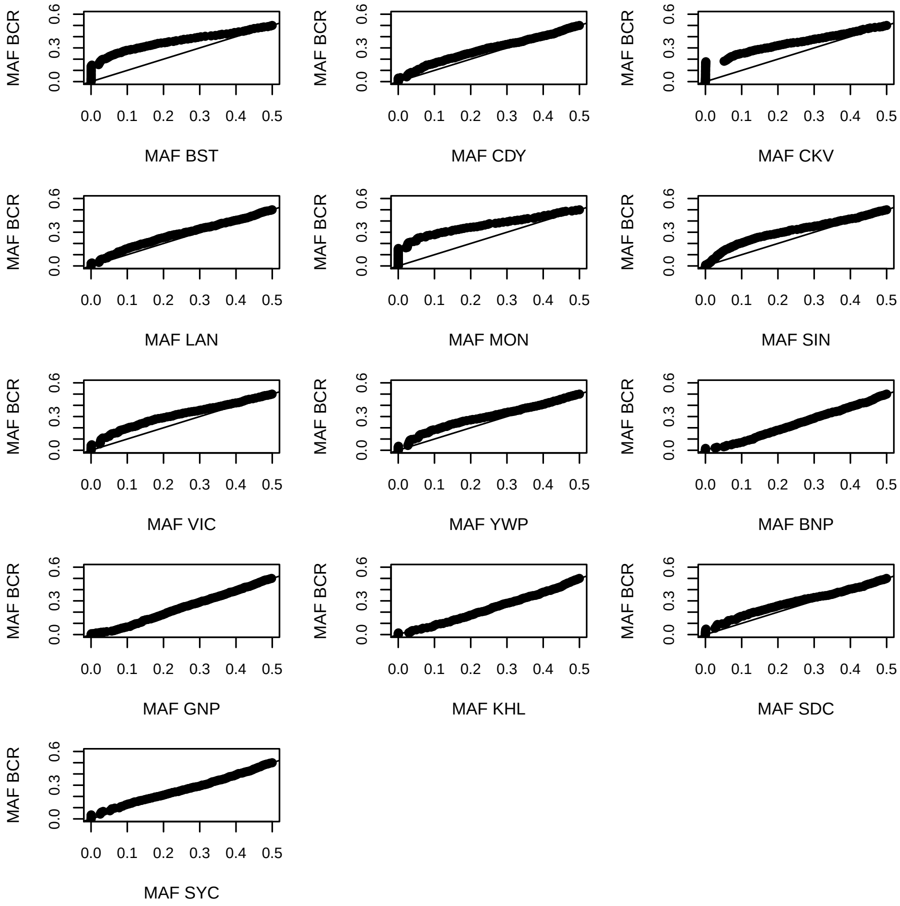

Supplementary Figure 10: Quantile-quantile plots show the distribution of minor allele frequency (MAF) for all pairs of ancestry informative SNPs (AIMs) for each population when compared to Bull Creek (BCR). Here, AIMs were defined as those SNPs with an allele frequency difference of at least 0.3 between *L. idas* and *L. melissa*. Population abbreviations and sample sizes are given in Supplementary Table 1.

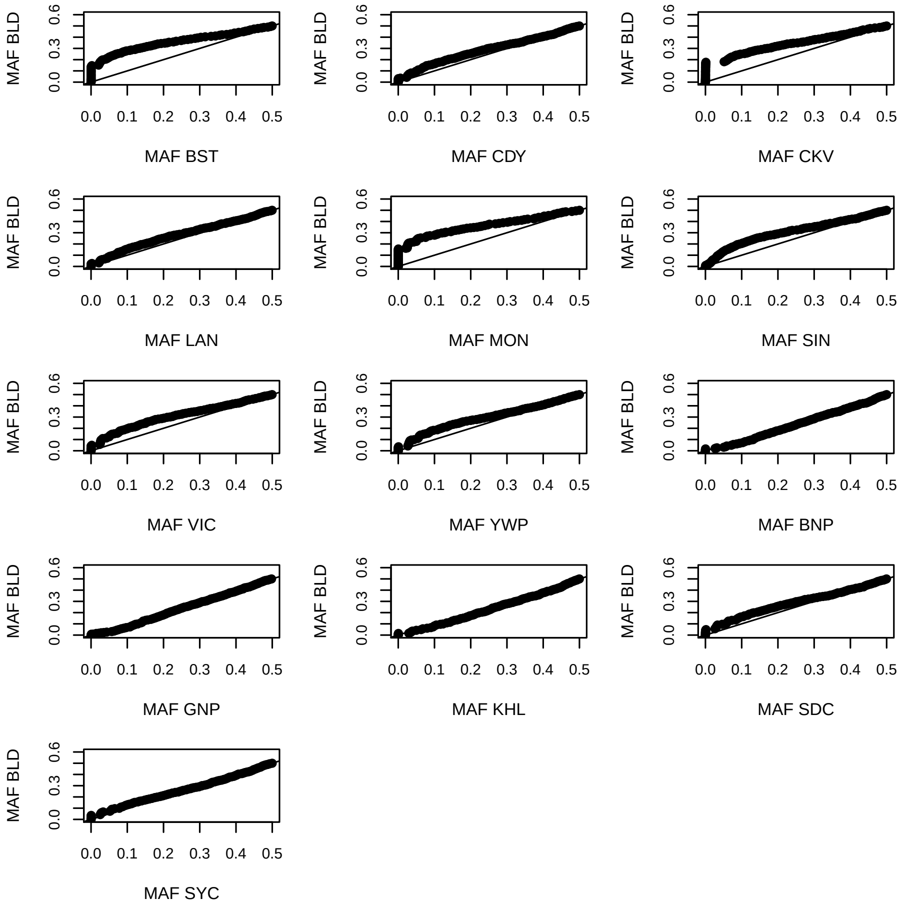

Supplementary Figure 11: Quantile-quantile plots show the distribution of minor allele frequency (MAF) for all pairs of ancestry informative SNPs (AIMs) for each population when compared to Bald Mountain (BLD). Here, AIMs were defined as those SNPs with an allele frequency difference of at least 0.3 between *L. idas* and *L. melissa*. Population abbreviations and sample sizes are given in Supplementary Table 1.

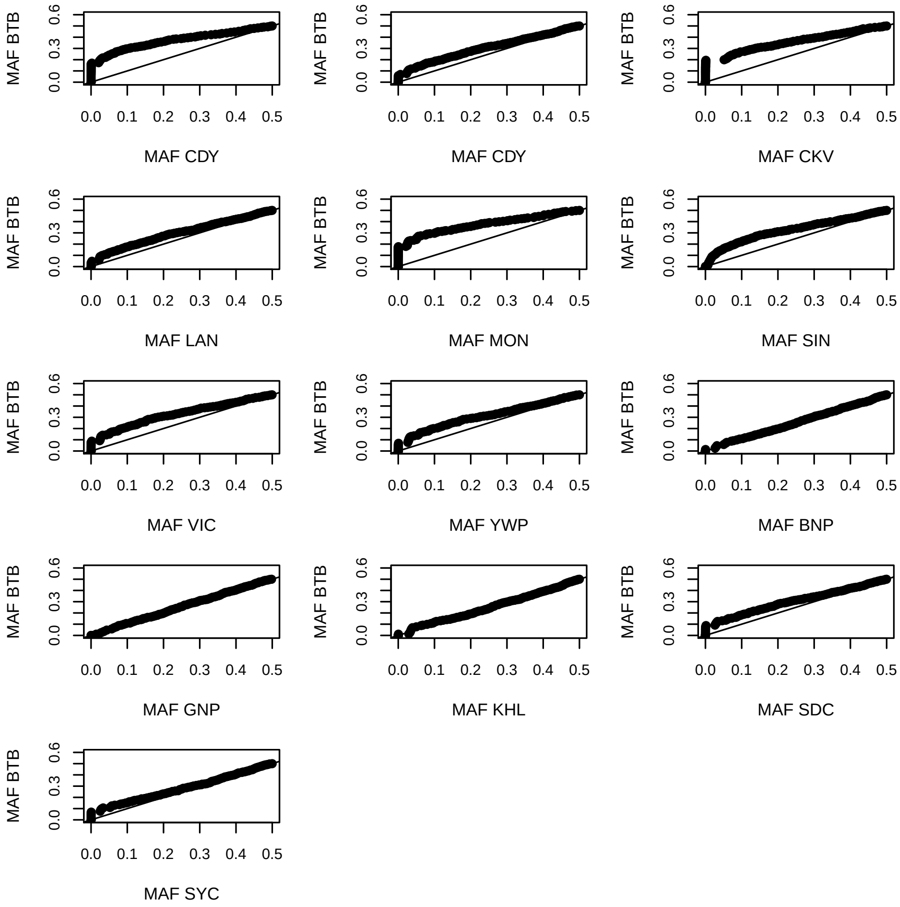

Supplementary Figure 12: Quantile-quantile plots show the distribution of minor allele frequency (MAF) for all pairs of ancestry informative SNPs (AIMs) for each population when compared to Blacktail Butte (BTB). Here, AIMs were defined as those SNPs with an allele frequency difference of at least 0.3 between *L. idas* and *L. melissa*. Population abbreviations and sample sizes are given in Supplementary Table 1.

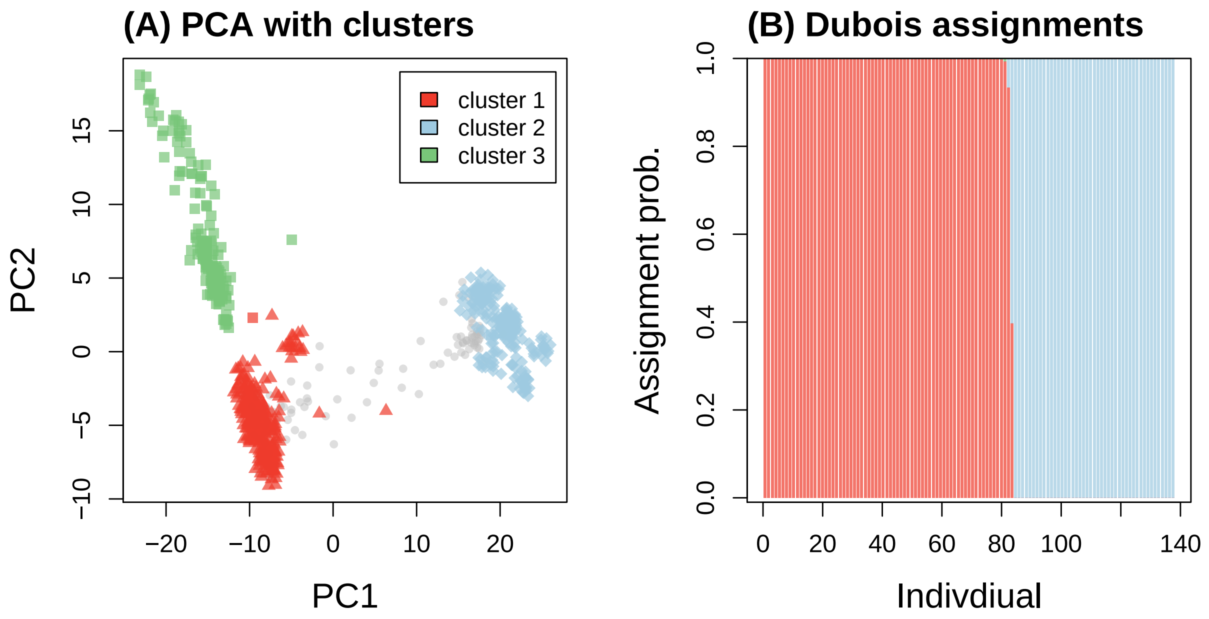

Supplementary Figure 13: Genetic clusters and genetic assignment of Dubois individuals. Panel (A) shows a results of a PCA for all $N = 835$ *Lycaeides*. Symbols denote nominal taxon (as in Figure 2): *L. idas* = square, *L. melissa* = diamond, Jackson Hole = triangle. Colors denote cluster assignment from k-means clustering. Dubois individuals (shown in light gray for reference) were not included in the k-means cluster analysis. Panel (B) shows assignment probabilities from discriminant function analysis of the genetic PCs (PC1 and PC2) for the Dubois individuals to each of the three genetic clusters from (A). Individuals are ordered based on their assignment probability to cluster 1 (which corresponds with Jackson Hole). No individuals had assignment probabilities > 0.01 to *L. idas*.

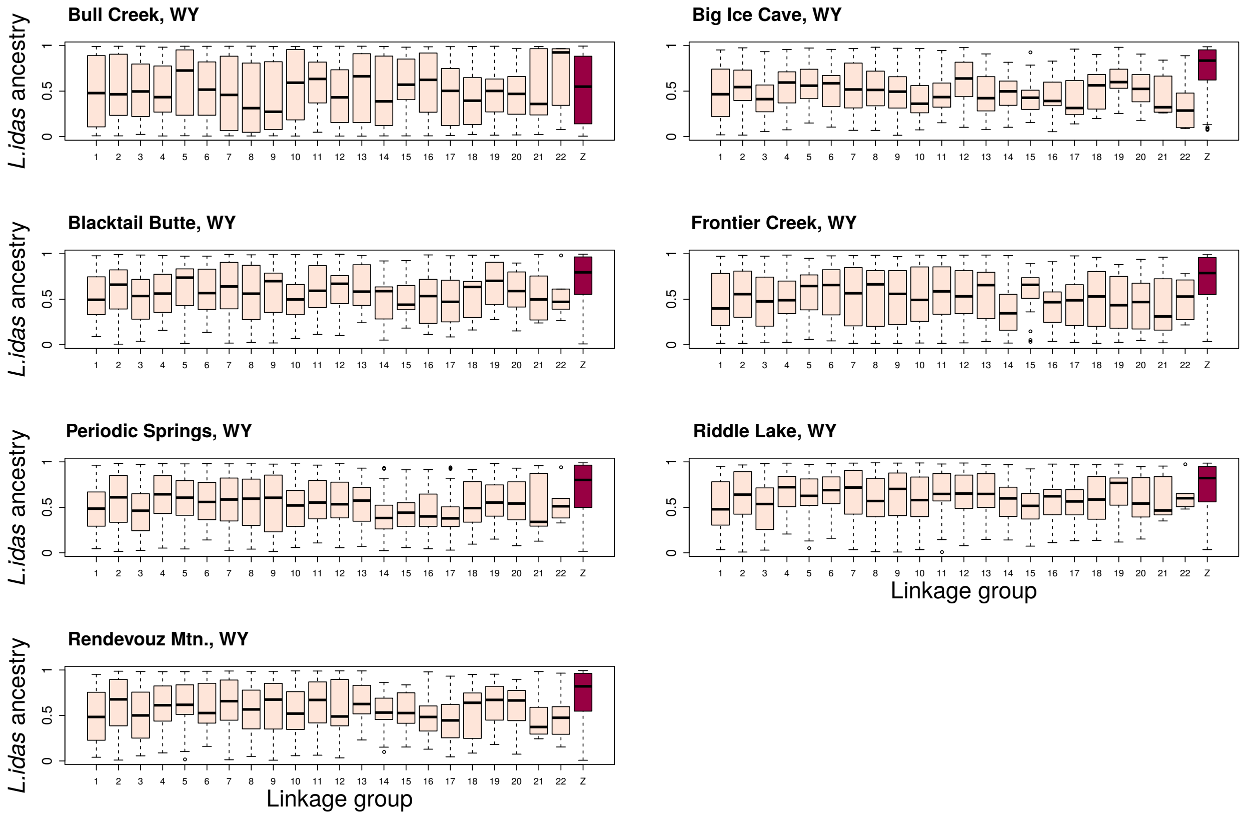

Supplementary Figure 14: Boxplots show the distribution of *L. idas* ancestry across ancestry informative SNPs (AIMs) for each linkage group in remaining Jackson Hole *Lycaeides* populations ($N = 1164$ loci and 212 butterflies from seven populations). Boxes denote the 1st and 3rd quartile with the median given by the midline; whiskers extend to the minimum and maximum value or 1.5× the interquartile range with points for more extreme values. The Z sex chromosome is shown in red.

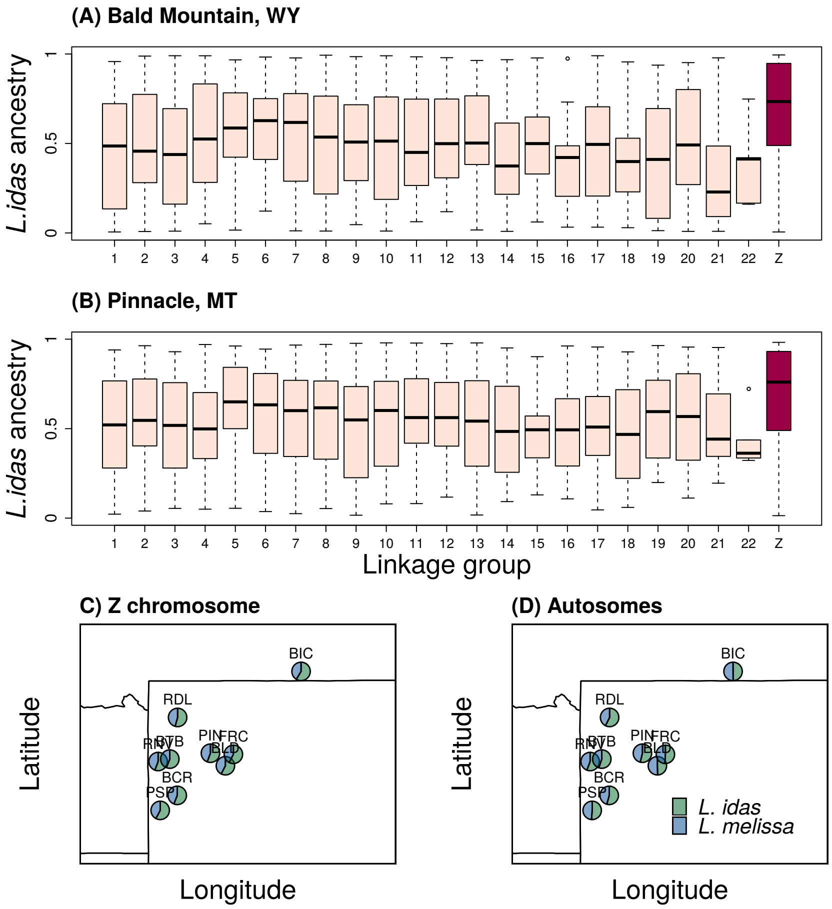

Supplementary Figure 15: Boxplots show the distribution of *L. idas* ancestry across ancestry informative SNPs (AIMs) for each linkage group in two representative Jackson Hole *Lycaeides* populations–Bald Mountain, WY (BLD; N = 48 male butterflies) (A) and Pinnacle, WY (PIN; N = 18 male butterflies) (B). Results are shown for male butterflies only. See Supplementary Figure 16 for additional populations. Boxes denote the 1st and 3rd quartile with the median given by the midline; whiskers extend to the minimum and maximum value or 1.5× the interquartile range with points for more extreme values. The Z sex chromosome is shown in red. Panels (C) and (D) show maps with pie charts reflecting the proportion of *L. idas* and *L. melissa* ancestry (mean) for the Z chromosome (C) and autosomes (D) for each of the nine populations (see Supplementary Table 1 for population IDs).

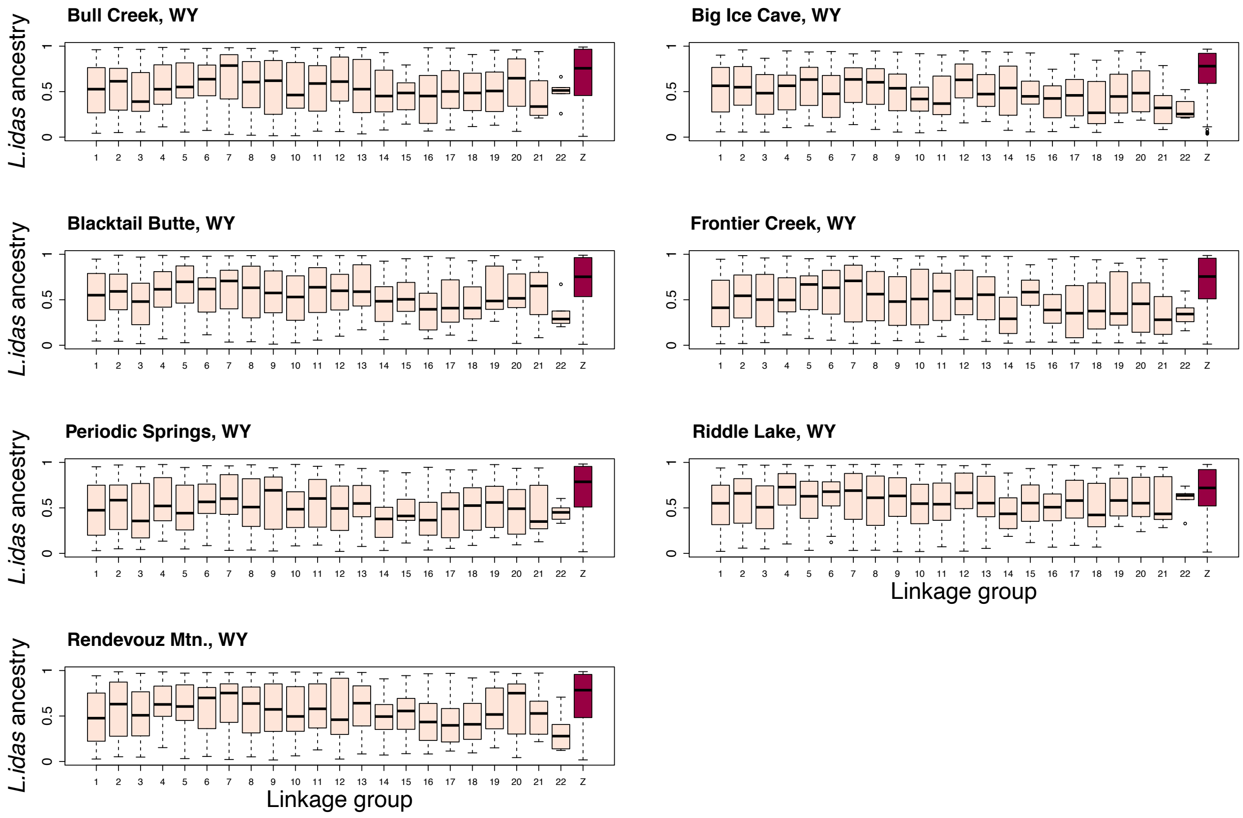

Supplementary Figure 16: Boxplots show the distribution of *L. idas* ancestry across ancestry informative SNPs (AIMs) for each linkage group in remaining Jackson Hole *Lycaeides* populations (N = 1164 loci and 115 male butterflies from seven populations). Results are shown for male butterflies only. Boxes denote the 1st and 3rd quartile with the median given by the midline; whiskers extend to the minimum and maximum value or 1.5× the interquartile range with points for more extreme values. The Z sex chromosome is shown in red.

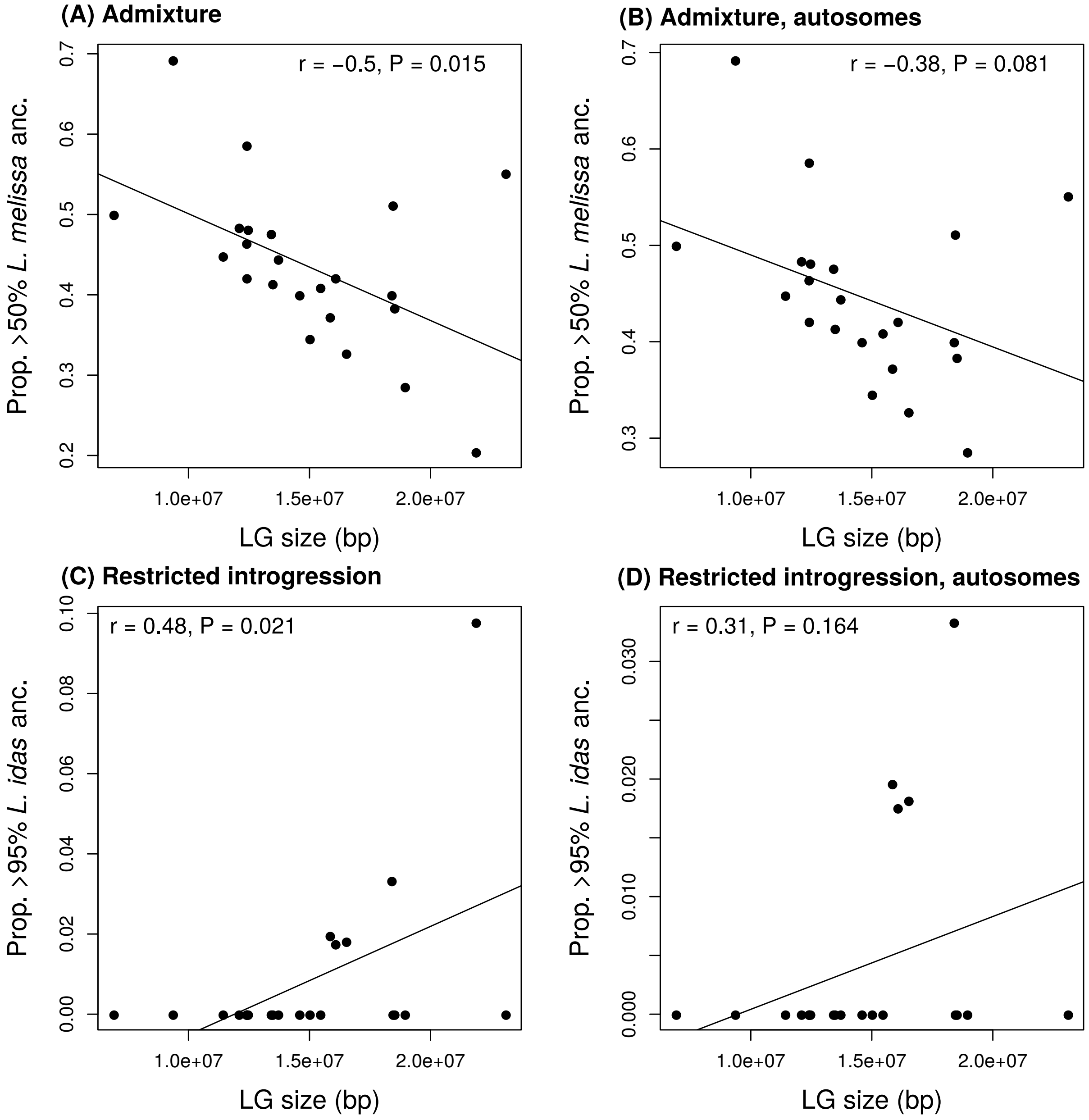

Supplementary Figure 17: Scatterplots show the relationship between chromosome size and admixture/introgression in Jackson Hole *Lycaeides*. Panels (A) and (B) show the proportion of loci with mean (across populations) ancestry from *L. melissa* > 50% (i.e., where *L. melissa* ancestry is more common than *L. idas* ancestry) for all chromosomes (A) or only the 22 autosomes (B). Panels (C) and (D) show the proportion of loci with mean (across populations) ancestry from *L. idas* > 95% (i.e., nearly fixed for *L. idas* ancestry across all Jackson Hole populations) for all chromosomes (C) or only the 22 autosomes (D). We report the Pearson r and two-sided P value for the correlation between ancestry and LG size, and give the best-fit line from a linear regression of ancestry on chromosome size.

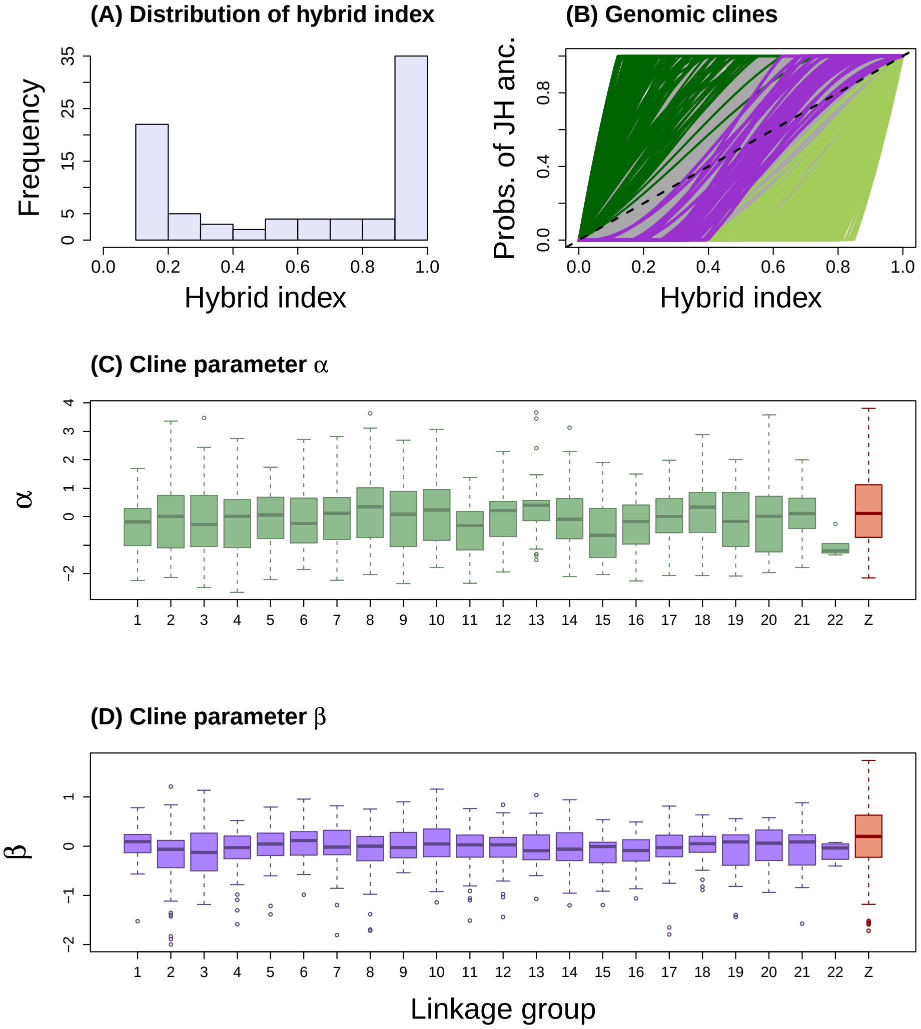

Supplementary Figure 18: Summary of the genomic clines analysis for male butterflies. (A) The histogram depicts the distribution of hybrid indices in the Dubois hybrid zone. (B) This plot shows estimated genomic clines for a subset of ancestry informative SNPs (AIMs). Each solid line gives the estimated probability of Jackson Hole (JH) ancestry for an AIM. Green lines denote cases of credible directional introgression (95% CIs for α that exclude zero) and purple lines denote credible cases of restricted introgression (95% CIs for $\beta > 0$) (gray lines denote clines not credibly different from the genome-average). The dashed line gives the null expectation based on genome-wide admixture. Twenty-seven ($\beta > 0$), 139 ($\alpha > 0$) and 272 ($\alpha < 0$) AIMs showed credible deviations from null expectations, and for $\beta > 0$ and $\alpha > 0$ these were over-represented on the Z chromosome ($\beta > 0$, number on Z = 25, x-fold enrichment = 4.48, one-sided $P < 0.001$; $\alpha > 0$, number on Z = 59, x-fold enrichment = 2.06, one-sided $P < 0.001$). Boxplots show the distribution of cline parameters α (C) and β (D) across loci for each linkage group (N = 1164 loci and 83 male butterflies). Boxes denote the 1st and 3rd quartile with the median given by the midline; whiskers extend to the minum and maximum value or 1.5× the interquartile range with points for more extreme values.

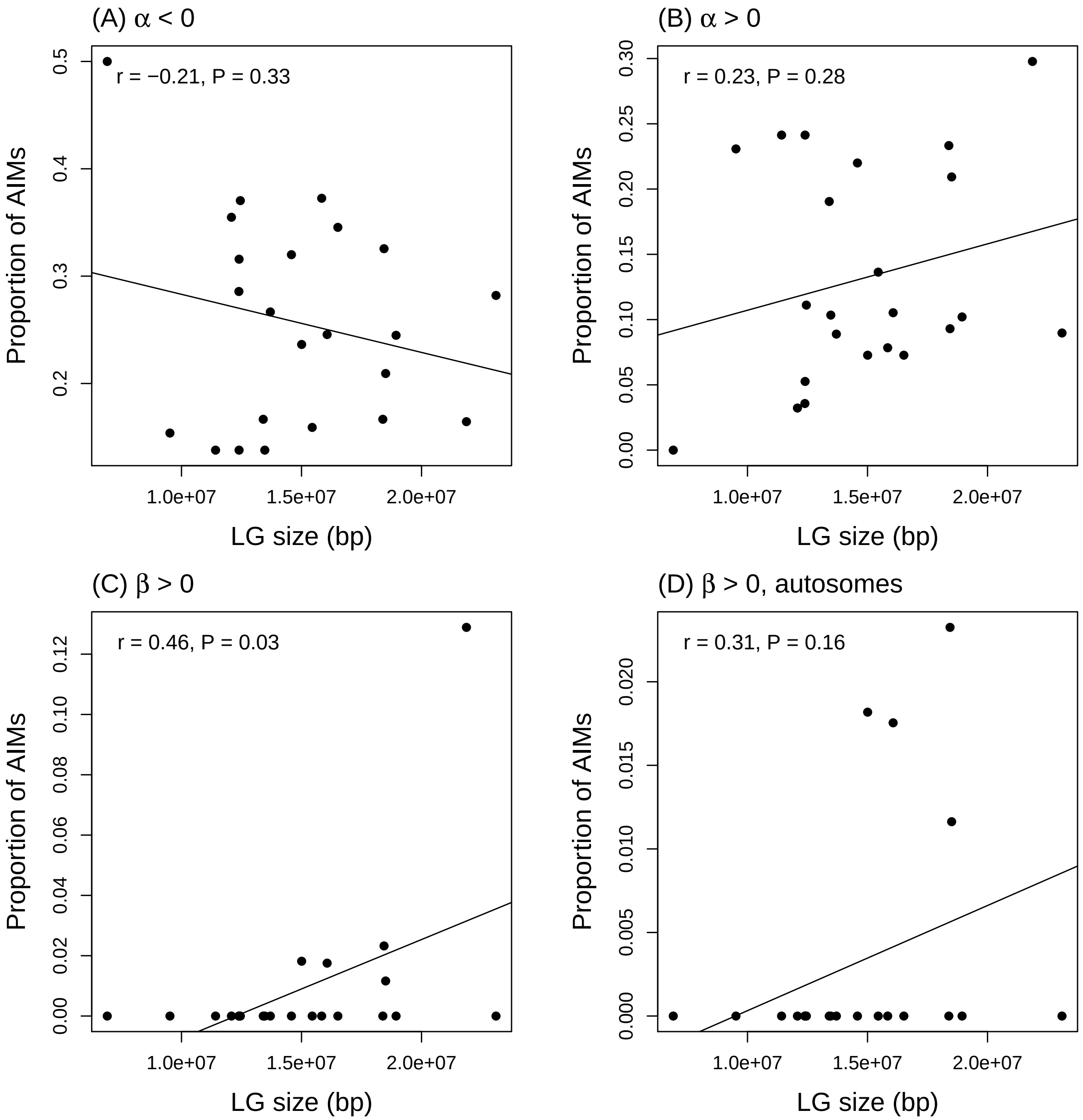

Supplementary Figure 19: Scatter plots depict the proportion of ancestry informative SNPs (AIMs) on each linkage group (LG) with patterns of introgression that deviate from genome-average expectations as a function of LG size. Panels (A-C) shows all chromosomes, whereas panel (D) considers only the 22 autosomes. Results are shown for $\alpha > 0$ (excess Jackson Hole *Lycaeides* introgression), $\alpha < 0$ (excess *L. melissa* introgression), and $\beta > 0$ (restricted introgression). The latter is shown with and without the Z chromosome. We report the Pearson correlation between the proportion of AIMs and LG size and associated two-sided P value, and give the best-fit line from a linear regression.

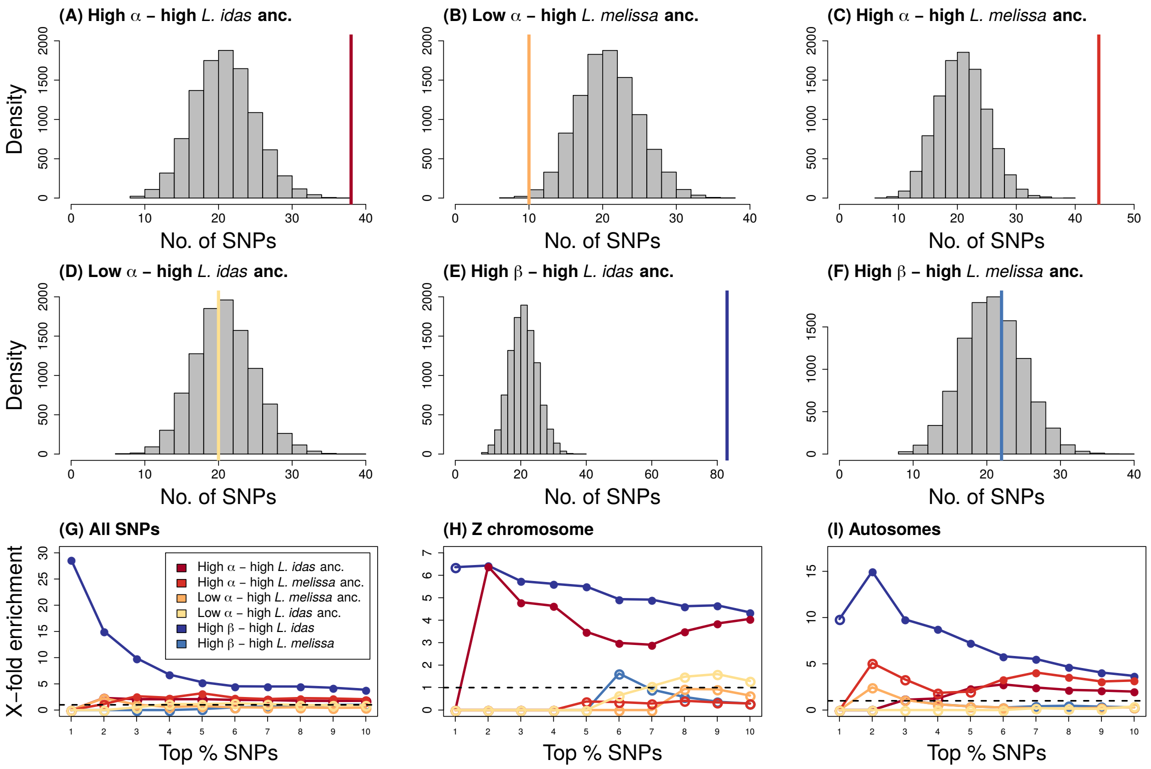

Supplementary Figure 20: Expected and observed numbers of SNPs with exceptional patterns of introgression in the Dubois hybrid zone and extreme ancestry frequencies in Jackson Hole *Lycaeides*. Here, results are shown for the 2126 SNPs with allele frequency differences of 0.2 or more between *L. idas* and *L. melissa*. Panels A-F show results when considering the top 10% of ancestry informative SNPs (AIMs) in each category. Histograms give null expectations from randomization tests and vertical solid lines show the observed number of AIMs exhibiting a given pattern. Comparisons shown are: directional introgression of Jackson Hole alleles (high α) and high *L. idas* ancestry (in Jackson Hole) (A), directional introgression of *L. melissa* alleles (low α) and high *L. melissa* ancestry (B), directional introgression of Jackson Hole alleles (high α) and high *L. melissa* ancestry (C), directional introgression of *L. melissa* alleles (low α) and high *L. idas* ancestry (D), restricted introgression (high β) and high *L. idas* ancestry (E), and restricted introgression (high β) and high *L. melissa* ancestry (F). Panels G-I show how these results are affected by considering different levels of stringency (i.e., by examining the most extreme 10% to the top 1% of AIMs with each pattern), and when considering only the Z chromosome (G) or only the autosomes (H). Here, circles denote the ratio of the observed to expected overlap from the null, and the circles are filled ($P \leq 0.05$) or not ($P > 0.05$) to denote whether the overlap is greater than expected by chance.

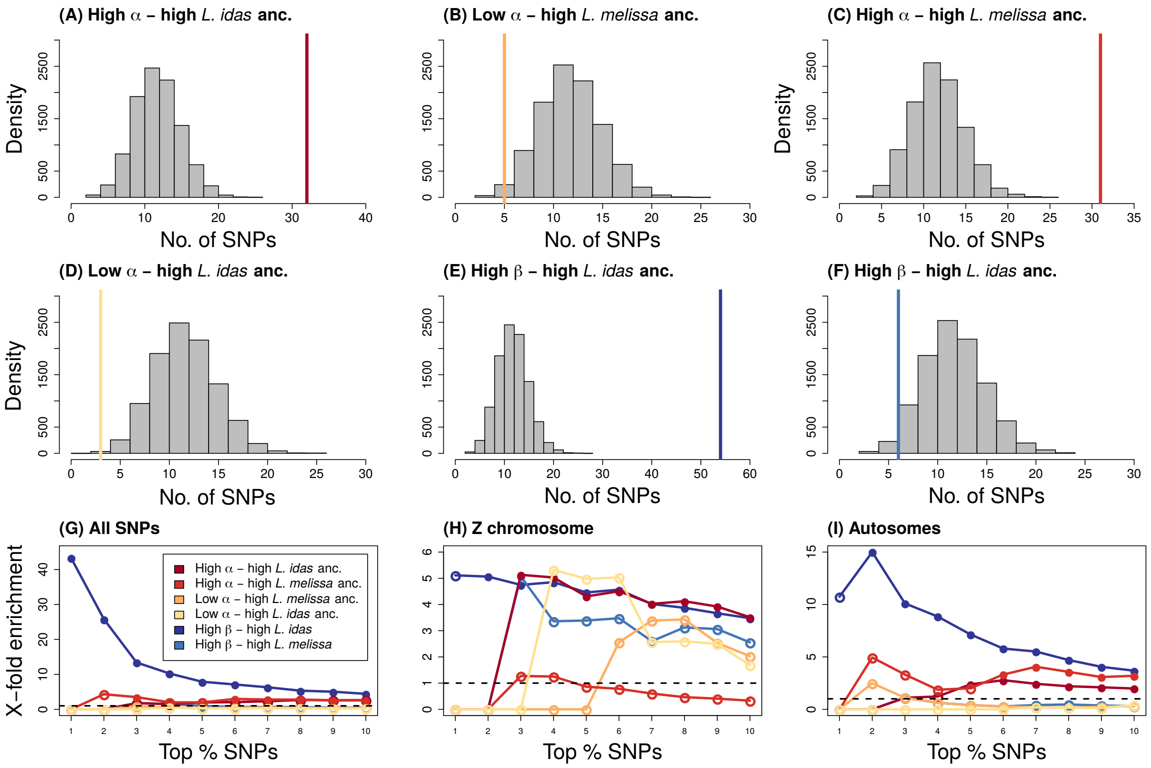

Supplementary Figure 21: Expected and observed numbers of SNPs with exceptional patterns of introgression in the Dubois hybrid zone and extreme ancestry frequencies in Jackson Hole *Lycaeides*. Here, results are shown for male butterflies only and for the 1164 SNPs with allele frequency differences of 0.3 or more between *L. idas* and *L. melissa*. Panels A-F show results when considering the top 10% of ancestry informative SNPs (AIMs) in each category. Histograms give null expectations from randomization tests and vertical solid lines show the observed number of AIMs exhibiting a given pattern. Comparisons shown are: directional introgression of Jackson Hole alleles (high α) and high *L. idas* ancestry (in Jackson Hole) (A), directional introgression of *L. melissa* alleles (low α) and high *L. melissa* ancestry (B), directional introgression of Jackson Hole alleles (high α) and high *L. melissa* ancestry (C), directional introgression of *L. melissa* alleles (low α) and high *L. idas* ancestry (D), restricted introgression (high β) and high *L. idas* ancestry (E), and restricted introgression (high β) and high *L. melissa* ancestry (F). Panels G-I show how these results are affected by considering different levels of stringency (i.e., by examining the most extreme 10% to the top 1% of AIMs with each pattern), and when considering only the Z chromosome (G) or only the autosomes (H). Here, circles denote the ratio of the observed to expected overlap from the null, and the circles are filled ($P \leq 0.05$) or not ($P > 0.05$) to denote whether the overlap is greater than expected by chance.

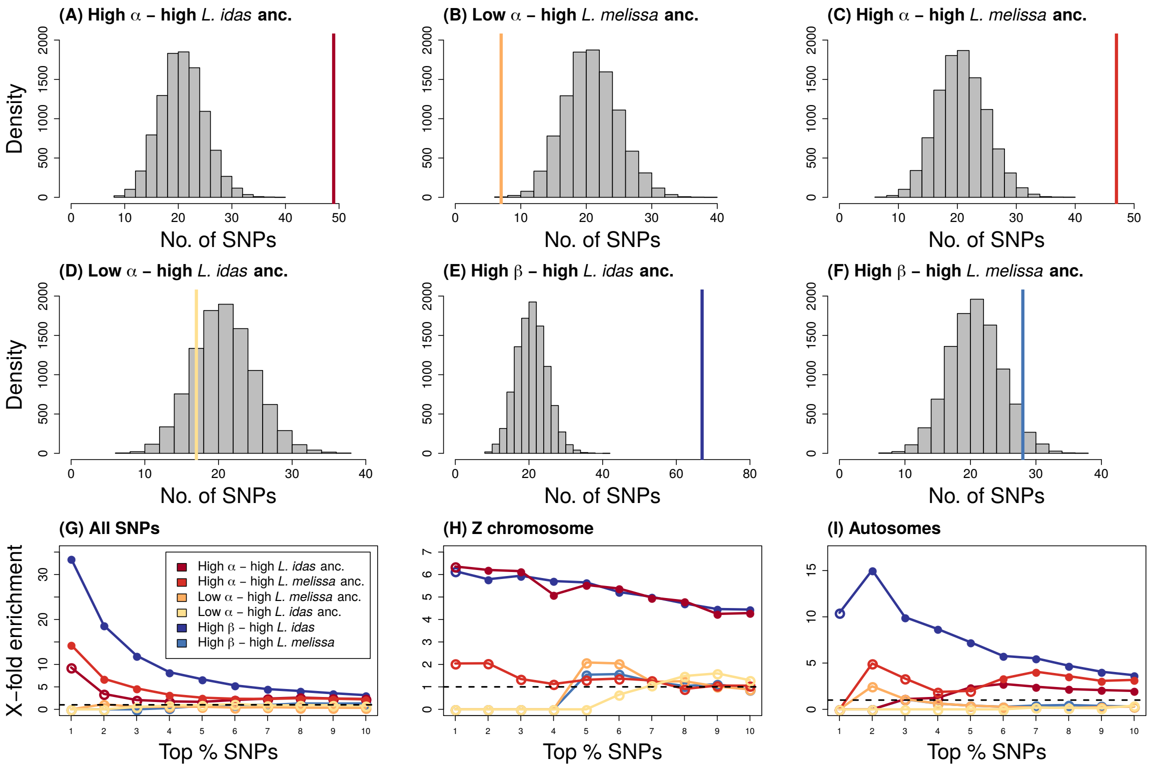

Supplementary Figure 22: Expected and observed numbers of SNPs with exceptional patterns of introgression in the Dubois hybrid zone and extreme ancestry frequencies in Jackson Hole *Lycaeides*. Here, results are shown for male butterflies only and for the 2126 SNPs with allele frequency differences of 0.2 or more between *L. idas* and *L. melissa*. Panels A-F show results when considering the top 10% of ancestry informative SNPs (AIMs) in each category. Histograms give null expectations from randomization tests and vertical solid lines show the observed number of AIMs exhibiting a given pattern. Comparisons shown are: directional introgression of Jackson Hole alleles (high α) and high *L. idas* ancestry (in Jackson Hole) (A), directional introgression of *L. melissa* alleles (low α) and high *L. melissa* ancestry (B), directional introgression of Jackson Hole alleles (high α) and high *L. melissa* ancestry (C), directional introgression of *L. melissa* alleles (low α) and high *L. idas* ancestry (D), restricted introgression (high β) and high *L. idas* ancestry (E), and restricted introgression (high β) and high *L. melissa* ancestry (F). Panels G-I show how these results are affected by considering different levels of stringency (i.e., by examining the most extreme 10% to the top 1% of AIMs with each pattern), and when considering only the Z chromosome (G) or only the autosomes (H). Here, circles denote the ratio of the observed to expected overlap from the null, and the circles are filled ($P \leq 0.05$) or not ($P > 0.05$) to denote whether the overlap is greater than expected by chance.

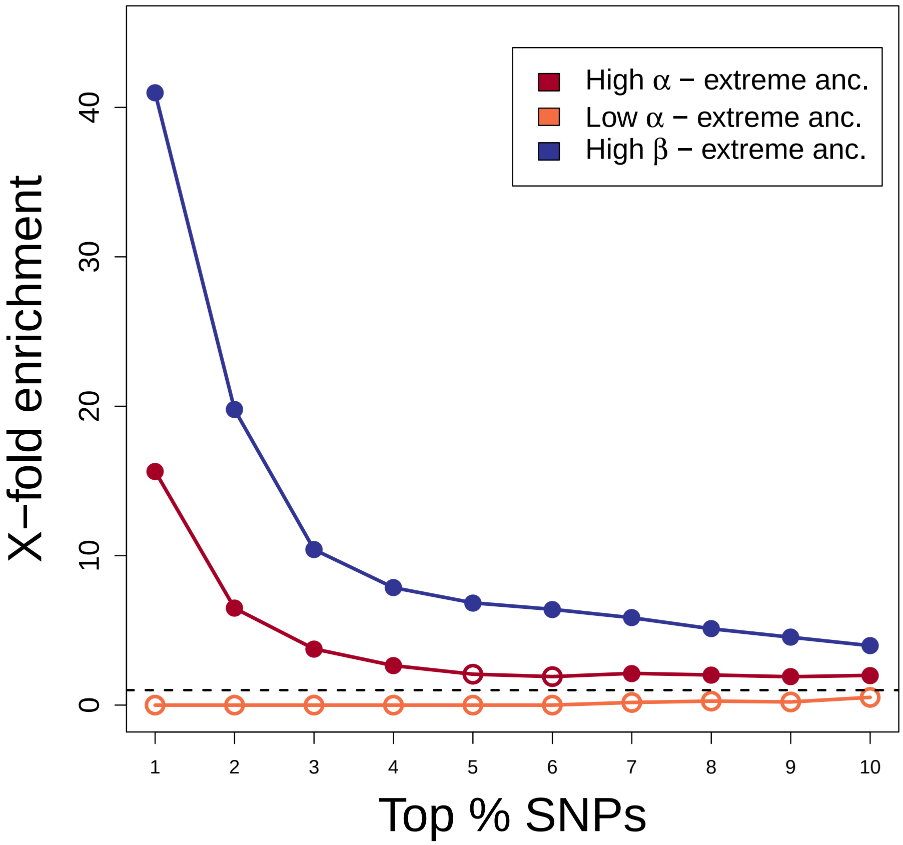

Supplementary Figure 23: Expected and observed numbers of SNPs with exceptional patterns of introgression in the Dubois hybrid zone and extreme ancestry frequencies in Jackson Hole *Lycaeides*. Comparisons shown are: directional introgression of Jackson Hole alleles (high α) and high *L. idas* or *L. melissa* ancestry (i.e., ancestry frequencies closest to 0 or 1), directional introgression of *L. melissa* alleles (low α) and high *L. idas* or *L. melissa* ancestry, and restricted introgression (high β) and high *L. idas* or *L. melissa* ancestry. Lines and points show these results are affected by considering different levels of stringency (i.e., by examining the most extreme 10% to the top 1% of ancestry informative SNPs (AIMs) with each pattern). Here, circles denote the ratio of the observed to expected overlap from the null, and the circles are filled ($P \leq 0.05$) or not ($P > 0.05$) to denote whether the overlap is greater than expected by chance.

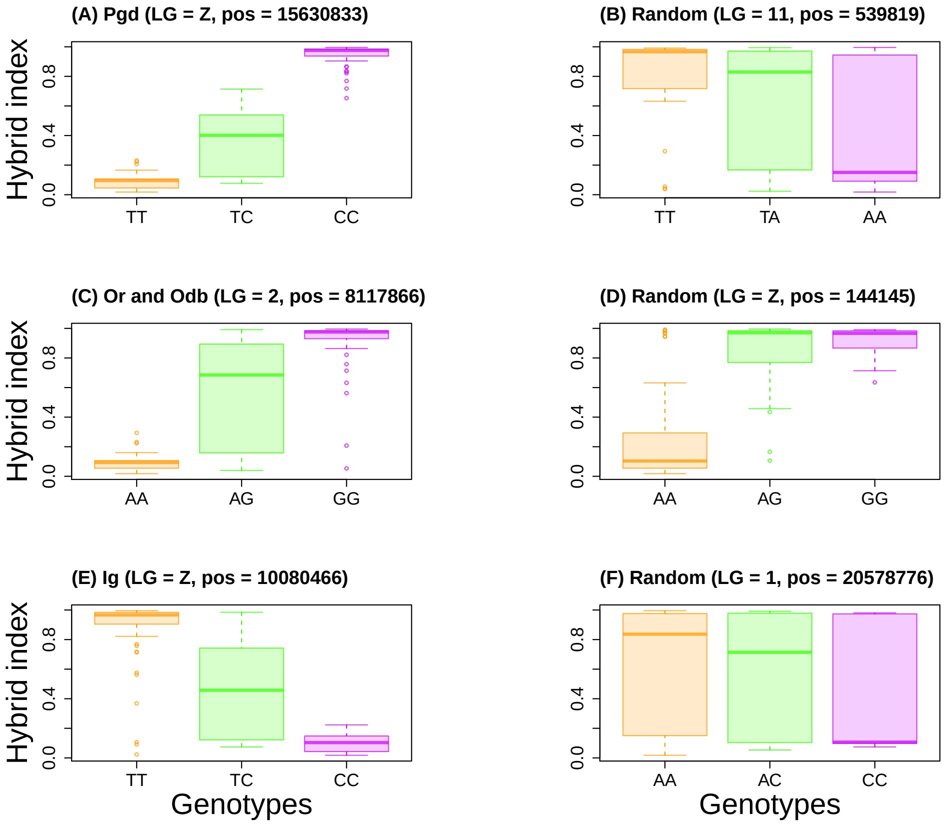

Supplementary Figure 24: Boxplots depict the distributions of hybrid indexes (from `bgc`) for individuals with each of three genotypes at each of six SNPs ($N = 115$ butterflies). Boxes denote the 1st and 3rd quartile with the median given by the midline; whiskers extend to the minimum and maximum value or 1.5$\times$ the interquartile range with points for more extreme values. SNPs shown include three from our candidate barrier loci–Pgd = phospogluconate dehydrogenase activity (A), Or = olfactory receptor activity and Odb = Odorant binding protein (C), and Ig = Immunoglobulin (E)–and three randomly selected SNPs (B, D, and F). The linkage group and position (in base pairs) of each SNP is given.

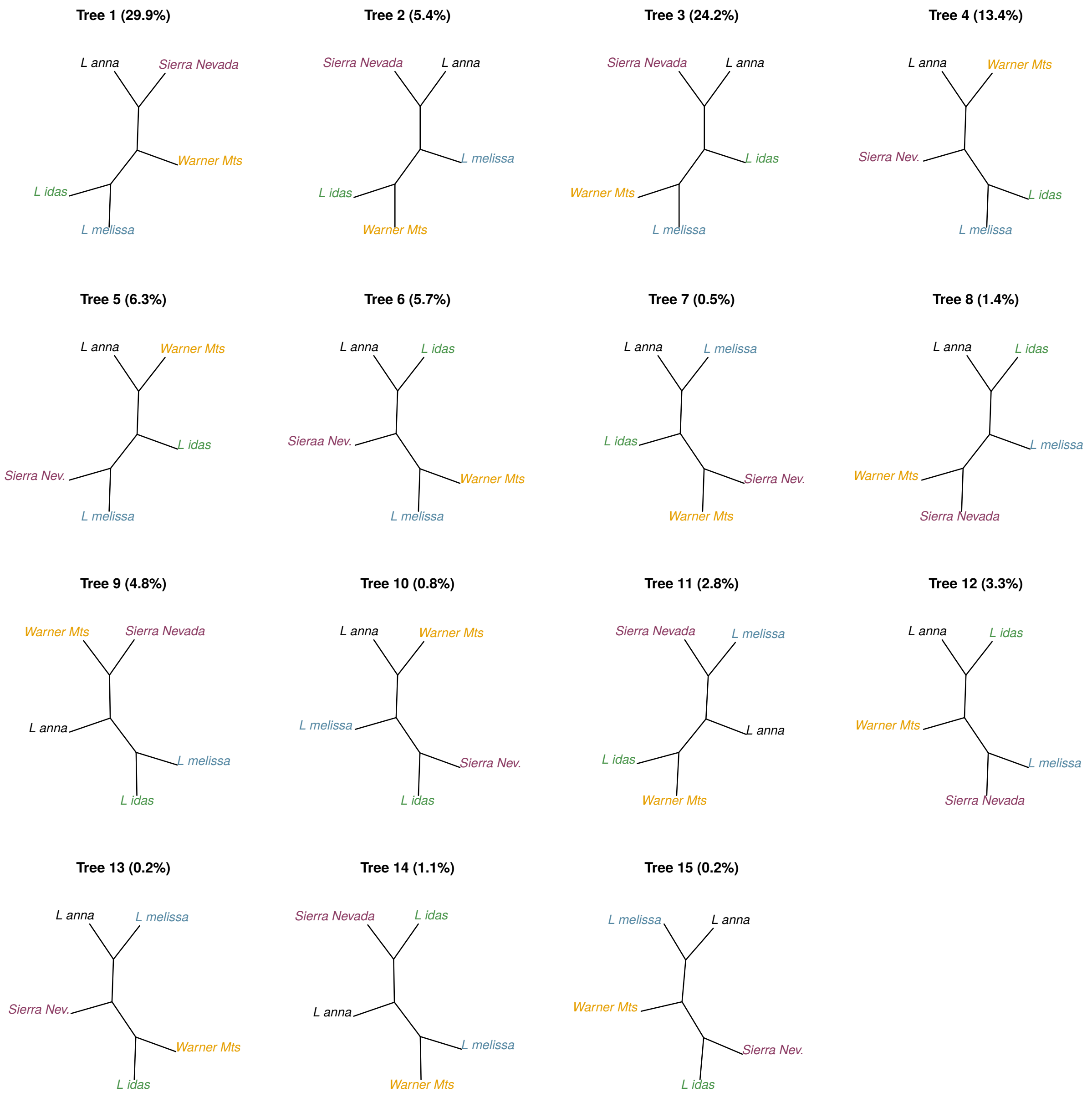

Supplementary Figure 25: Plot of all 15 observed, unrooted tree topologies. Unrooted maximum likelihood trees were inferred from non-overlapping 1000 SNP windows designated on each linkage group. Numbers in parentheses give the percent of trees that matched each topology.

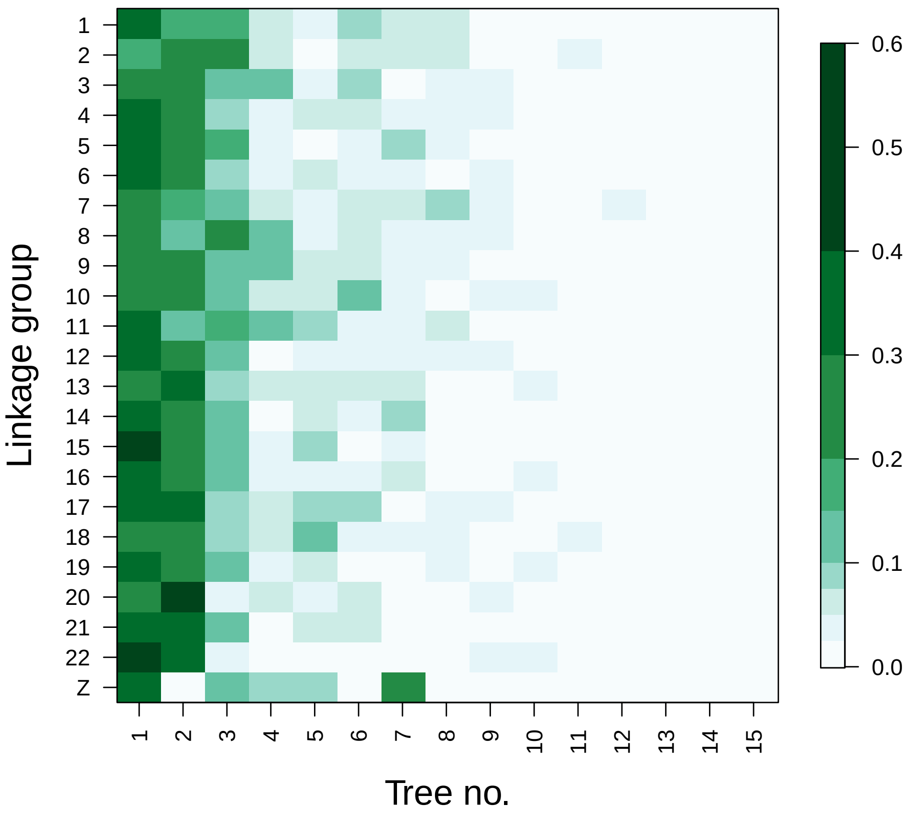

Supplementary Figure 26: This heatmap shows the proportion of unrooted trees matching each of 15 tree topologies for each linkage group. Unrooted maximum likelihood trees were inferred from non-overlapping 1000 SNP windows designated on each linkage group. Trees 1, 2 and 7 correspond to the topologies shown in Figure 6 panels (C), (D) and (E), respectively.

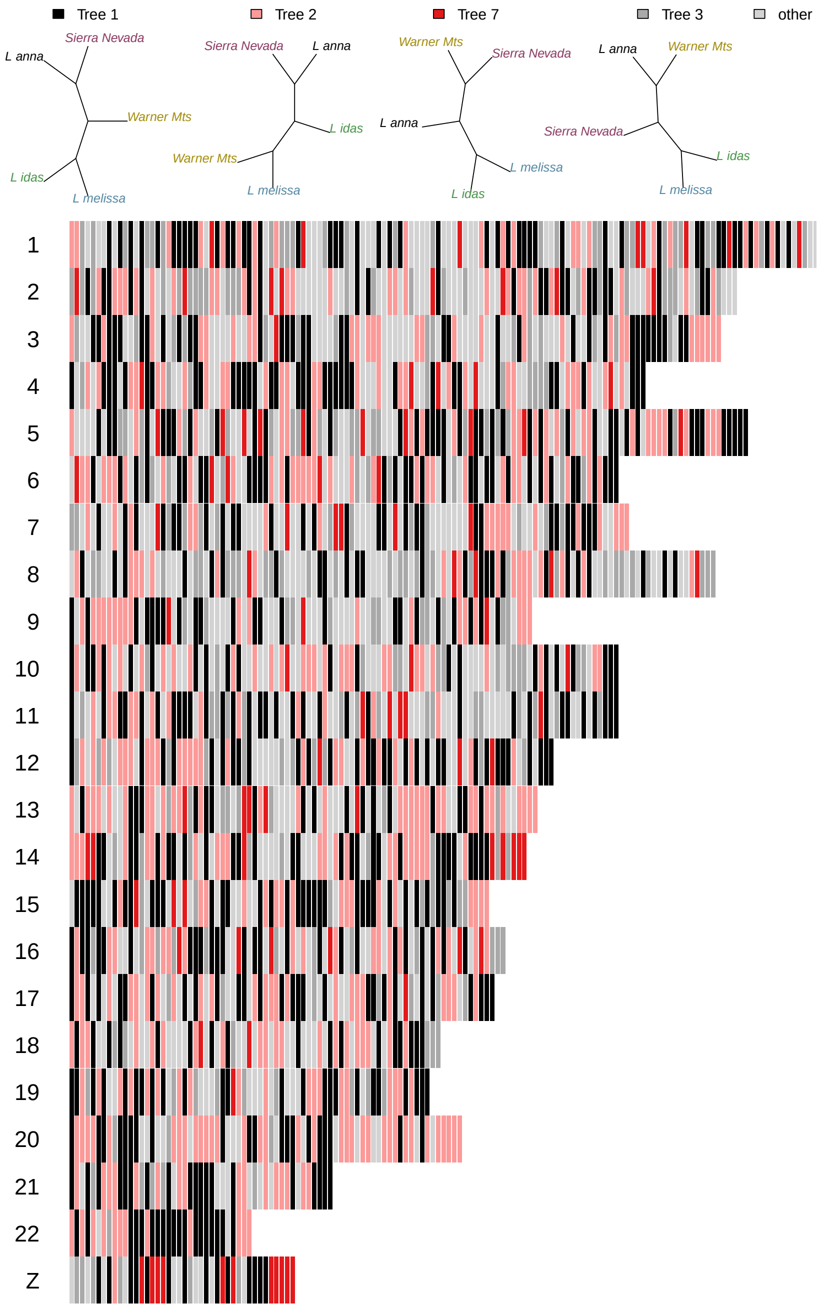

Supplementary Figure 27: Colored bars denote tree topologies as they vary along linkage groups. Unrooted maximum likelihood trees were inferred from non-overlapping 1000 SNP windows designated on each linkage group. Trees 1, 2 and 7 correspond to the topologies shown in Figure 6 panels (C), (D) and (E), respectively. Colored bars denote the topology for each 1000 SNP window and these are ordered according physical position along linkage groups. Light gray denotes all topologies except for the trees shown in the legend. Tree topologies exhibit autocorrelations along chromosomes. For example, autocorrelations for tree topology 7 (the restricted introgression tree) on the Z chromosome are greater than expected by chance at lags of one ($r = 0.41$, $P = 0.002$) and two windows ($r = 0.43$, $P = 0.001$).

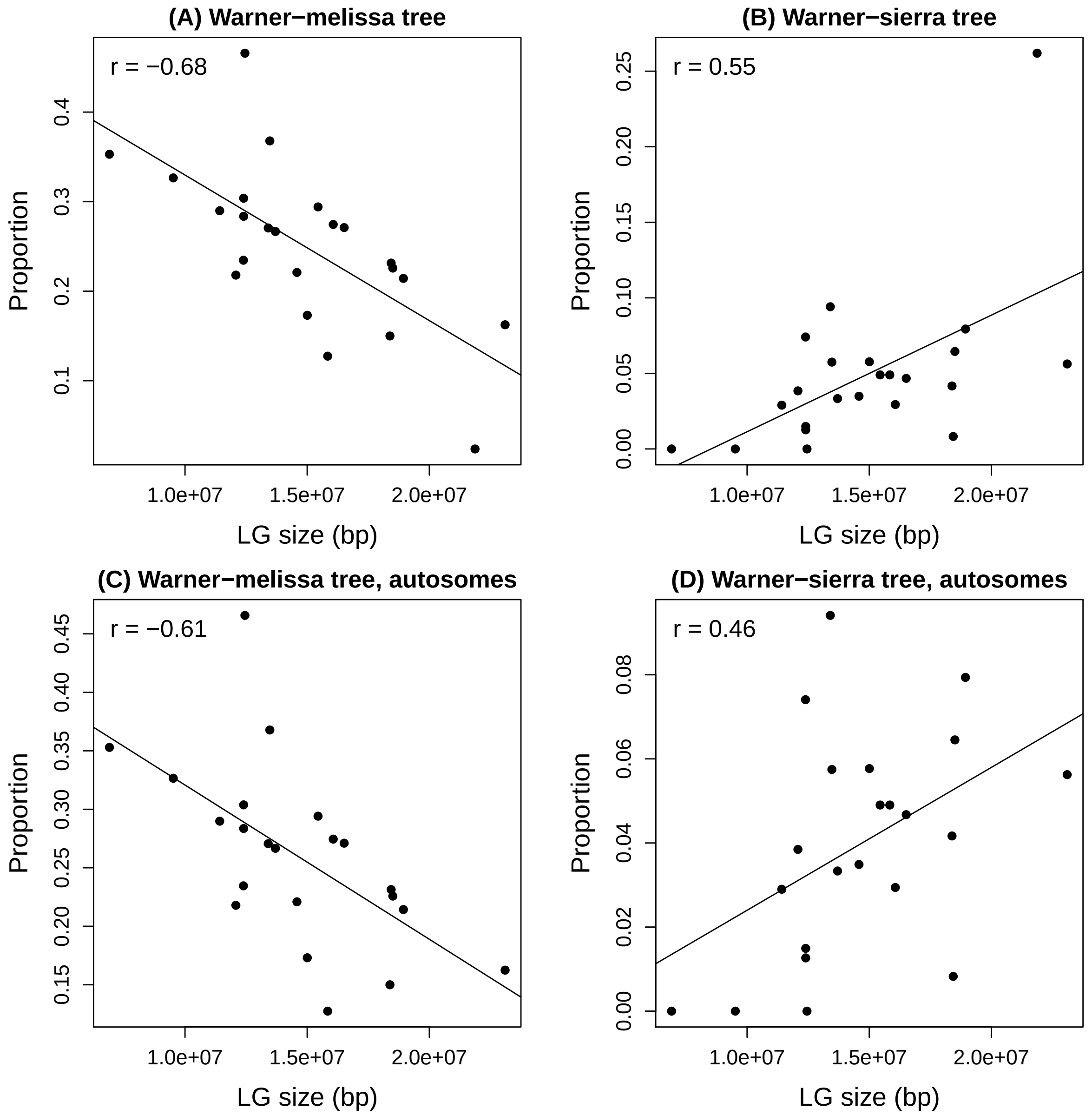

Supplementary Figure 28: Scatter plots depict the proportion of tree topologies in 1000 SNP windows for each linkage group (LG) as a function of linkage group size in base pairs (bp). Panels (A) and (C) show the proportion of trees with the Warner-*melissa* topology (tree 2), whereas panels (B) and (D) who the proportion of trees with the Warner-sierra topology (tree 7). The top panels show all chromosomes, whereas the bottom panels consider only the 22 autosomes (i.e., these exclude the Z chromosome). We report the Pearson correlation between the proportion of the tree topology and LG size, and give the best-fit line from a linear regression; two-sided P values for the effect of LG size were <0.001 (A), 0.006 (B), 0.003 (C), and 0.033 (D).

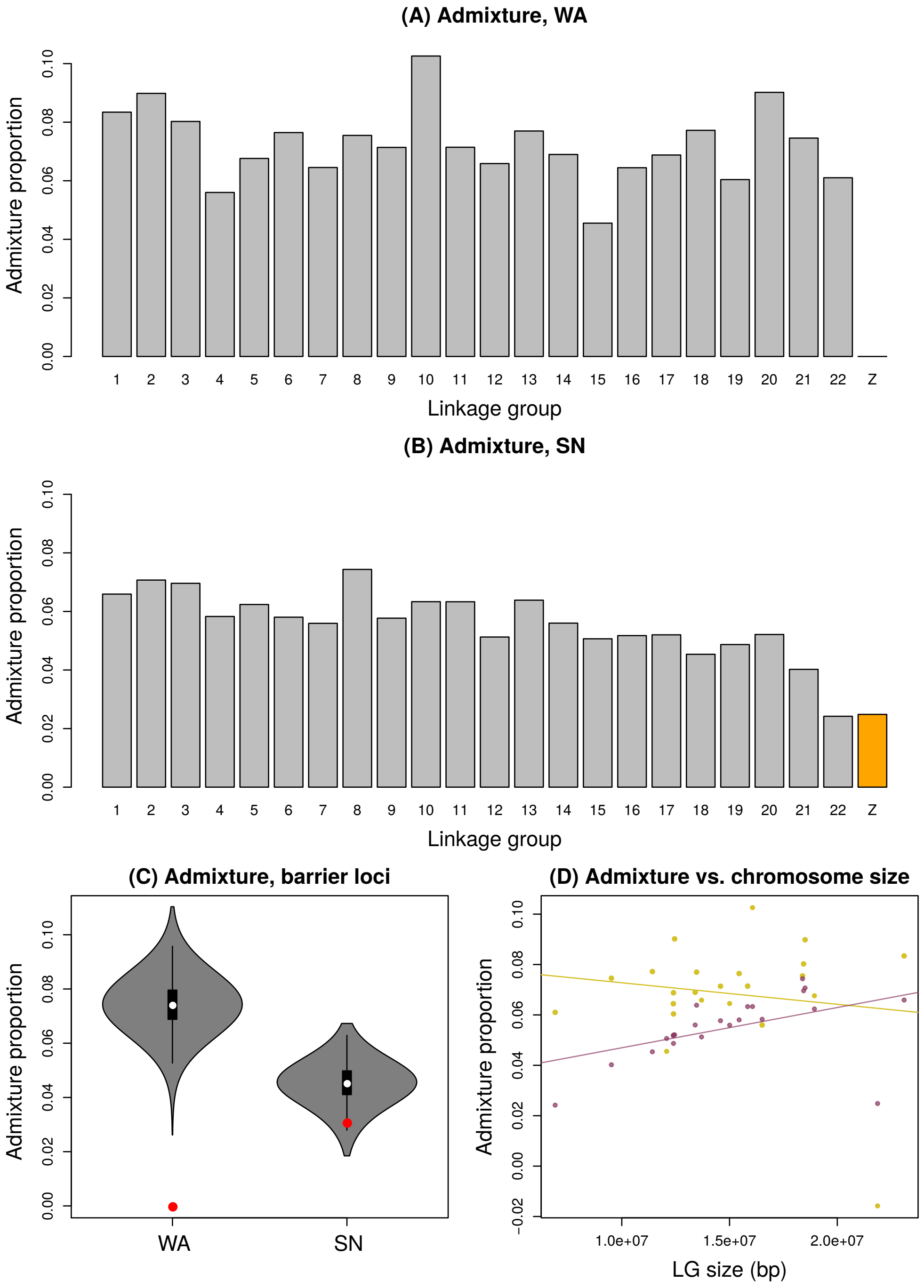

Supplementary Figure 29: Summary of admixture proportions (f_d estimates) from the phylogenomic analyses. Panels (A) and (B) show admixture proportions for four-taxon trees with the Warner mountains = WA (A) or Sierra Nevada = SN (B) populations by linkage group. Violin plots in panel (C) show the distribution of expected (from a randomization test) admixture proportions for the 51 barrier loci. Here, white dots denote the median of the null, black boxes denote the 1st and 3rd quartile, vertical bars extend to the minimum and maximum null values or 1.5× the interquartile range, and kernel densities show the full null distribution. Red dots show the observed values. Panel (D) shows the relationship between chromosome (LG = linkage group) size and admixture proportion for the Warner mountains (red) (β = -8.5e^{-10}, two-sided P = 0.50, r^2 = 0.02), and Sierra Nevada (gold) (β = 1.6e^{-9}, two-sided P = 0.02, r^2 = 0.23). Points show individual values and the lines show the best fit relationship.

Supplementary References

[1] Gompert, Z. *et al.* Admixture and the organization of genetic diversity in a butterfly species complex revealed through common and rare genetic variants. *Molecular Ecology* **23**, 4555–4573 (2014). URL http://dx.doi.org/10.1111/mec.12811.
