# Supplementary material for: Recent hybrids recapitulate ancient hybrid outcomes
Source: Nat Commun. 2020 May 1;11:2179. doi: 10.1038/s41467-020-15641-x (PMC7195404; doi:10.1038/s41467-020-15641-x)
Supplement: Supplementary file 3 — Reporting Summary [file 41467_2020_15641_MOESM3_ESM.pdf]

nature research

Corresponding author(s): Zachariah Gompert

Last updated by author(s): Oct 9, 2019

Reporting Summary

Nature Research wishes to improve the reproducibility of the work that we publish. This form provides structure for consistency and transparency in reporting. For further information on Nature Research policies, see Authors & Referees and the Editorial Policy Checklist .

Statistics

For all statistical analyses, confirm that the following items are present in the figure legend, table legend, main text, or Methods section.

n/a	Confirmed	
☐	☒	The exact sample size (n) for each experimental group/condition, given as a discrete number and unit of measurement
☐	☒	A statement on whether measurements were taken from distinct samples or whether the same sample was measured repeatedly
☐	☒	The statistical test(s) used AND whether they are one- or two-sided<br>*Only common tests should be described solely by name; describe more complex techniques in the Methods section.*
☒	☐	A description of all covariates tested
☐	☒	A description of any assumptions or corrections, such as tests of normality and adjustment for multiple comparisons
☐	☒	A full description of the statistical parameters including central tendency (e.g. means) or other basic estimates (e.g. regression coefficient) AND variation (e.g. standard deviation) or associated estimates of uncertainty (e.g. confidence intervals)
☐	☒	For null hypothesis testing, the test statistic (e.g. F, t, r) with confidence intervals, effect sizes, degrees of freedom and P value noted<br>*Give P values as exact values whenever suitable.*
☐	☒	For Bayesian analysis, information on the choice of priors and Markov chain Monte Carlo settings
☒	☐	For hierarchical and complex designs, identification of the appropriate level for tests and full reporting of outcomes
☐	☒	Estimates of effect sizes (e.g. Cohen's d, Pearson's r), indicating how they were calculated

Our web collection on statistics for biologists contains articles on many of the points above.

Software and code

Policy information about availability of computer code

Data collection	No software/computer code was used for data collection.
Data analysis	We used the following published, freely available computer programs:<br>- maker (version 2.31.10) = genome annotation pipeline<br>-LepBase (version 4.0) = reference sequences for genome annotation<br>- repeatscout (version 1.0.5) = genome annotation, repeat identification<br>- repeatmasker (4.0.7) = genome annotation, repeat identification<br>- trimgalore (version 2.6.6) = adapter trimming of RNA seq data for genome annotation<br>- snap (version 2006-07-08) = genome annotation, gene prediction<br>- augustus (version 3.3) = genome annotation, gene prediction<br>- trinity (version 2.6.6) = do novo transcriptome assembly<br>- blastp (version 2.3.0+) = genome annotation, align maker output to proteins<br>- interproscan (5.32-71.0) = genome annotation, protein ids<br>-mummer (3.2) = whole genome comparative alignment for designating linkage groups<br>- bwa (version 0.7.17) = DNA sequence alignment<br>- samtools (version 1.5) = parsing DNA sequence alignment<br>- bcftools (version 1.6) = variant calling<br>- entropy (version 1.2) = Bayesian inference of genotypes and admixture proportions<br>- estpEM (version 0.1) = maximum likelihood inference of population allele frequencies<br>- bgc (version 1.04b) = Bayesian inference of genomic clines<br>- popanc (version 0.1) = estimation of population ancestry frequencies<br>- eems (version 0.0.0.9000) = inference of effective migration rates<br>- JAGS (4.3.0) = Bayesian inference with Gibbs sampling, isolation-by-distance

- GATK (version 3.5) = variant calling for whole genome resequence data
- RAxML (version 8.2.9) = maximum likelihood estimation of phylogenetic trees
- R (version 3.5.1) = general environment for statistical computing
- rjags (4.8) = R interface with JAGS
- phytools (version 0.6.60) = R package for phylogeny manipulation/visualization
- ape (version 5.2) = R package for phylogeny manipulation/visualization

Additional custom perl and R scripts and example usage and analyses of the programs above are available on GitHub, https://github.com/karwaan/Ancient-and-contemporary-hybrids_ms/tree/master and Dryad (doi:10.5061/dryad.76hdr7ssw)

For manuscripts utilizing custom algorithms or software that are central to the research but not yet described in published literature, software must be made available to editors/reviewers. We strongly encourage code deposition in a community repository (e.g. GitHub). See the Nature Research guidelines for submitting code & software for further information.

Data

Policy information about availability of data

All manuscripts must include a data availability statement. This statement should provide the following information, where applicable:

- Accession codes, unique identifiers, or web links for publicly available datasets
- A list of figures that have associated raw data
- A description of any restrictions on data availability

DNA sequence data that support the findings of this study have been deposited in the NCBIs SRA with accession codes PRJNA577236 [\url{https://www.ncbi.nlm.nih.gov/bioproject/PRJNA577236}] and PRJNA432816 [\url{https://www.ncbi.nlm.nih.gov/sra/?term=PRJNA432816}]). The \emph{L. melissa} genome assembly, annotation, RNA sequence data used for the annotation and SNP variant file that also support the findings of this study have been deposited in Dryad (doi:10.5061/dryad.j3tx95x9d [\url{https://doi.org/10.5061/dryad.j3tx95x9d}]). Publicly available databases that support the findings of this study are UNIPROT/SWISSPROT [\url{http://www.uniprot.org}] and LepBase version 4.0 [\url{http://lepbase.org/}]. The source data underlying Figures 2-6 are provided as a Source Data file. A Code Availability statement is also included in the manuscript.

Field-specific reporting

Please select the one below that is the best fit for your research. If you are not sure, read the appropriate sections before making your selection.

☐ Life sciences ☐ Behavioural & social sciences ☒ Ecological, evolutionary & environmental sciences

For a reference copy of the document with all sections, see nature.com/documents/nr-reporting-summary-flat.pdf

Ecological, evolutionary & environmental sciences study design

All studies must disclose on these points even when the disclosure is negative.

Study description	We took advantage of natural hybridization in North American Lycaeides butterflies to test the consistency of genome composition between a contemporary hybrid zone and multiple, ancient hybrid lineages that have progressed towards genome stabilization. Using population genomic data, we verified the existence of a contemporary hybrid zone between L. melissa and Jackson Hole Lycaeides; the latter is an ancient hybrid lineage derived from L. melissa and L. idas. We then used a mixture of partial and whole genome sequence data to compare patterns of introgression in this hybrid zone to the genomic mosaic of ancestry in Jackson Hole Lycaeides, and to additional ancient hybrid lineages in the Sierra Nevada and Warner mountains, and thereby quantified the consistency of hybrid genome composition across these cases. We thus used the contemporary hybrid zone as a window on the evolutionary process and asked whether similar or very different processes operated during the origin and establishment of multiple, ancient hybrid lineages.
Research sample	We analyzed partial genome sequences from 835 Lycaeides butterflies from 23 populations in western North America: eight L. melissa populations (N = 306 butterflies), five L. idas populations (N = 176 butterflies), 9 Jackson Hole Lycaeides populations (N = 326 butterflies) and the Dubois hybrid zone (N = 115 butterflies). The sequence data from 643 of these butterflies was previously described in a study of admixture across the Lycaeides species complex (Gompert et al. 2014). Data from 192 of the butterflies were generated for the current study, and this includes many (but not all) of the Dubois individuals. Specific locations (latitude and longitude) and sample sizes for each population are provided in the manuscript (Table S1).
Sampling strategy	Butterflies were collected from sites known to harbor Lycaeides based on our 20+ years of field work with this system. A sufficient number of butterflies were collected to obtain reasonably precise estimates of population allele frequencies (20+ for most populations, resulting in 40+ gene copies). These are equal to or greater than what is commonly seen in contemporary population genomic studies. In some cases, sample sizes were dictated by collecting permits.
Data collection	DNA sequence data were generated by on an Illumina HiSeq 2500 (100 bp, single-end reads) by the Genome Sequencing and Analysis Facility at the University of Texas (Austin, TX).
Timing and spatial scale	Data (butterfly samples) were collected from 23 populations (spatial locations) across western North America between 2008 and 2016.
Data exclusions	No data were excluded from the analyses.
Reproducibility	We repeated most analyses with different cut-offs for defining ancestry informative markers and with all butterflies or only males (the homogametic sex). We generally obtained results consistent with/similar to those described in the main text. These

reproducibility/sensitivity analyses are described in full in the manuscript.

Randomization	Samples were not allocated into treatments, rather in this population genomic study samples were associated with the populations from which they were collected.
Blinding	Population genomic analyses were conducted with computational algorithms that were generally ignorant of the population from which an sample was collected except in cases where this information was a component of the analysis. IDs for specific individuals were never a component of the analyses.

Did the study involve field work? ☒ Yes ☐ No

Field work, collection and transport

Field conditions	Samples were collected by hiking to field sites during June, July and August of 2008 to 2016. Samples were collected using insect nets. Field work was conducted between 10 am and 5 pm, that is, during the time of day where butterflies are most active. Samples were mostly collected on warm days with full or partial sun.
Location	We collected 835 Lycaeides butterflies from 23 populations in western North America: eight L. melissa populations (N = 238 butterflies), five L. idas populations (N = 176 butterflies), 9 Jackson Hole Lycaeides populations (N = 306 butterflies) and the Dubois hybrid zone (N = 115 butterflies). Specific locations (latitude and longitude) and sample sizes for each population are provided in the manuscript (Table S1).
Access and import/export	All samples were collected from within the USA (no import/export). Samples were collected by driving/hiking to field sites. Samples collected from Yellowstone National Park GRand Grand Teton National Park were collected in accordance with US national park study permits YELL-05924 and GRTE-00285, respectively.
Disturbance	Minimal to no disturbance was caused by this study.

Reporting for specific materials, systems and methods

We require information from authors about some types of materials, experimental systems and methods used in many studies. Here, indicate whether each material, system or method listed is relevant to your study. If you are not sure if a list item applies to your research, read the appropriate section before selecting a response.

Materials & experimental systems

n/a	Involved in the study	
☒	☐	Antibodies
☒	☐	Eukaryotic cell lines
☒	☐	Palaeontology
☐	☒	Animals and other organisms
☒	☐	Human research participants
☒	☐	Clinical data

Methods

n/a	Involved in the study	
☒	☐	ChIP-seq
☒	☐	Flow cytometry
☒	☐	MRI-based neuroimaging

Animals and other organisms

Policy information about studies involving animals; ARRIVE guidelines recommended for reporting animal research

Laboratory animals	No laboratory animals were involved.
Wild animals	Butterflies were collected in the field with insect nets and then transported to the lab or field station in glassine envelopes. Butterflies were killed by freezing at -20C.
Field-collected samples	Butterflies were collected in the field with insect nets and then transported to the lab or field station in glassine envelopes. Butterflies were killed by freezing at -20C. Butterflies were then stored at -80C and maintained at that temperature until DNA extraction.
Ethics oversight	The study was restricted to insects, thus no 'animals' (i.e., no vertebrates) were used and ethics approval was not required.

Note that full information on the approval of the study protocol must also be provided in the manuscript.
