# Supplementary material for: Recent hybrids recapitulate ancient hybrid outcomes
Source: Nat Commun. 2020 May 1;11:2179. doi: 10.1038/s41467-020-15641-x (PMC7195404; doi:10.1038/s41467-020-15641-x)
Supplement: Supplementary file 2 — Peer Review File [file 41467_2020_15641_MOESM2_ESM.pdf]

Reviewers' comments:

Reviewer #1 (Remarks to the Author):

The paper addresses an important and interesting question, comparing an older hybrid zone with contemporary hybridization. Overall, I think this is a great study that will be broadly interesting, the analyses are thorough, and the paper is very well written. I think the authors did an excellent job of distilling very complex analyses into a concise manuscript. I am very excited about this paper and I enjoyed reading it, but I do have concerns about how some of the results are interpreted.

The introduction and discussion only considered selection as a mechanism for consistent genomic patterns between ancient and recent hybrids. I think ignoring the recent literature on the role of recombination, gene density, and other structural features on patterns of divergence and shared ancestry is major omission. Structural features of the genome can also lead to shared ancestry tracks, for instance introgressed segments can persist in regions of the genome where there is low recombination. The authors do allude to this on Lines 298 and 299, but I think this is relevant to the discussion about the Jackson Hole/Dubois populations as well.

I don't think "ancient" is the right word for the Jackson Hole hybrids. Generally, the term ancient hybridization is used for cases where hybridization is found between allopatric taxa or taxa where one lineage is extinct. What is the evidence that there is no recent hybridization in Jackson Hole or that the Jackson Hole hybrid populations have progressed towards genome stabilization? I agree that the analyses here suggest the Dubois population is more recent admixture than other Jackson sampling sites and I think the comparisons of these populations is fundamentally interesting. But to me, admixture in the Jackson Hole populations is more consistent with adjacent/intermixed populations that experience periods of contact and backcrossing in a patchy environment (Gompert et al. 2010), rather than an ancient stabilized hybrid lineage.

As it is currently presented, the discussion and presentation of the admixture results is a bit misleading. Fig 1 shows an idealized version of hybrid indices for an ancient hybrid lineage and recent hybridization, but only the hybrid indices of Dubois population (recent hybridization) are presented in the main text (e.g. Fig 3A). The distribution of hybrid indices for Jackson Hole is presented in a slightly different format in Fig S1, but does not match the predictions of ancient hybrid lineage presented in Fig 1. Jackson Hole hybrids have hybrid indices close to L. idas, with evidence of admixture up to ~0.20 from L. melissa, which I would interpret as backcrossing of rare hybrids into L. idas, with little gene flow from L. idas allopatric populations. Indeed, in a prior study the ancestry of Jackson Hole populations was so close to L. idas, that there was no evidence of admixture using treemix and structure plots generally showed the same patterns between L. idas and Jackson Hole, with slightly more L. melissa ancestry in Jackson Hole (Gompert et al. 2014, Figs 4, 5, 6). The relative amount of admixture for Jackson Hole populations are not that different from the admixture in what is defined as pure L. melissa populations (Fig S1, this manuscript). I think all of this could be made clearer is some version of Fig S1 were included in the main manuscript.

I think the description of the results of the PCA is also a bit misleading. There is not a clear distinction between Jackson Hole hybrids and L. idas. There are L. idas individuals clustered with Jackson Hole, and vice versa. While there are variants that distinguish these overall populations, the same pattern can be seen in modern humans or any other species that has population structure and isolation by distance. Given the past interpretations of the patchy population structure of these sampling sites, the manuscript should include explicit measures of IBD. Finally, while I agree it is likely that the Dubois population is a product of hybridization between L. melissa and Jackson Hole (based on the location of the sites), I don't see how that can be firmly established given that there are both Jackson Hole and L. idas individuals clustering with some Dubois individuals in the PCA? Are there additional analyses that support this? Overall, I think the manuscript should be more cautious in interpreting the PCA.

All that being said, I think this is a matter of presentation and I don't think that being more cautious in the interpretations would decrease the impact or the appeal of the manuscript. Regardless of how the Jackson Hole hybrids are described, it is still a very interesting and exciting study to compare ancestry in these hybrids relative to new Dubois populations.

Minor comments:

I think the patterns of consistently restricted introgression on the Z chromosome is indeed interesting, though it isn't particularly exceptional (Line 300). The discussion of factors underlying restricted Z introgression should also mention linkage (modeled in Muirhead and Presgraves, The American Naturalist, 2016).

Line 52: "genome collisions" is a bit much (also Line 86, 314).

Line 78: citation?

Line 102: The cluster for Jackson Hole is not particularly tight, and if other populations of Lycaeides were included in the PCA, then Jackson Hole populations cluster with L. idas, as presented in Gompert et al. 2014 (Fig 1B).

Fig 1C: Maybe indicate that this is the hypothesized relationship among these populations?

Fig 2: The populations on the map could be related to the PCA through either a gradient of colors or shapes. The shapes/colors could also correspond to the PC scores in Fig 2C. Color blind readers can't distinguish this color palette, and especially in this figure, would miss critical information. The colors should be changed.

Fig 5 is an excellent summary of results, but it could be easier to read if it were a bit bigger, especially the text?

Fig S2: If this is mean LD shouldn't there be standard deviation or error estimates? Maybe I am misunderstanding how mean LD is calculated.

Line 239-242: It is exciting to see a cold tolerance/diapause gene in the list of candidates, especially given the difference in diapause between these two species.

Line 306-310: It could be said that any study of speciation links macro and microevolutionary processes (in fact, that is how I define the study of speciation), and to be fair many studies make those links much more explicitly than this study, and in a comparative phylogenetic framework. I think this is overselling.

Line 410: typo? "to gene model"

Line 414: 'partial genome sequences' sounds like whole genome data. It might be clearer to identify these data as GBS. (also on Line 79)

Reviewer #2 (Remarks to the Author):

Chaturvedi et al. studied the genomic composition of hybrid-origin lineages of Lycaeides butterflies using a large dataset and a newly-assembled genome. Hybridization provides an exceptional opportunity to study the impacts of selection on the genome and its role in the origin of species. The study system and dataset used here are well suited to address these questions. The main finding of

this study is that genomic regions showing extreme ancestry of one or the other parental species in an older (Jackson Hole) hybrid lineage show significant overlap with those showing non-neutral patterns of ancestry (either excess introgression or barrier-like patterns) in a younger (Dubois) hybrid lineage. This highlights an important role for selection in speciation.

Overall I think this is a well-conducted study and a well written paper with results of broad interest. Most of my concerns are about providing more information and interpretation to improve clarity.

1. Developing expectations
The major obstacle for me in getting my head around this study is the fact that the two main focal lineages, Jackson Hole (JH) and Dubois (DBS), are not independent realizations of distinct hybridization events. Rather, the former lineage is thought to be a progenitor of the latter (line 77). While the non-independence is not a fundamental problem, it does alter our expectations. For example in Figure 1, we see how the genomes of ancient hybrids might have largely stabilized, whereas those of contemporary hybrids can be variable, but if we place this example in the context of the present study, and assume that blue represents L. melissa ancestry and gray represents L. idas ancestry, then the combination of ancestral and contemporary hybrids shown in Figure 1 would not be possible in the case of JH and DBS: the contemporary hybrids have idas ancestry in parts of their genomes where neither of their parental lineages (JH and melissa) could have contributed it. My point here is not about this schematic figure per se, but that the nuances of this situation need to be better laid out for the reader in the text early on. For example, in parts of the genome where JH is fixed for melissa ancestry do we even expect to be able to distinguish JH and melissa ancestry in the DBS population? (See my next point).

I also found the order of the first two results sections a bit odd. It is difficult to interpret the clines in the DBS population without first understanding the composition of JH. For example, reading that only 10.9 and 3.5% of the genome of JH is fixed for ancestry of each parental species did change my view of the whole story (again, perhaps Figure 1 is a bit misleading here). I also thought that before seeing Figure S6 it would be useful to see an equivalent figure showing how JH frequencies compare to those in its parental populations. Unless there is good reason not to, I suggest the authors consider placing the section on JH ancestry first in the results.

2. AIMs for JH
The Ancestry Informative Markers used for bgc in DBS are said to be defined based on frequencies in idas and melissa (line 123), but they have (understandably) been applied using JH as a reference population. Given that JH is itself partly of melissa origin, do these AIMs provide sufficient power to distinguish JH and melissa ancestry in DBS in all parts of the genome? For example if there is less melissa ancestry on the Z than on autosomes in JH, then perhaps there is also more power on the Z to detect JH ancestry in DBS. If so, is the finding of greater JH introgression on Z in DBS still valid?

My above concern was somewhat alleviated when I later saw that AIMs in regions of excess melissa ancestry in JH also show evidence of excess introgression in DBS, but as noted above, I am puzzled about how it is possible to distinguish melissa and JH ancestry in such regions. I expect this apparent paradox and its potential implications for systematic biases will concern other readers too, so it should be directly addressed in the text.

3. Introgression from JH
Again related to the above - of the three significant overlaps in Figure 6, I was surprised by the overlap between AIMs showing evidence of introgression into DBS and regions of extreme melissa ancestry in JH. Since DBS received melissa-like variation from both parental species, why should the JH alleles introgress and replace those from melissa?

4. Overlaps
Is there a reason that AIMs showing evidence of introgression (positive alpha) are compared

separately to regions that show either excess idas or melissa ancestry in JH, whereas AIMs showing barrier like patterns (positive beta) are compared with a combined 'extreme' category?

5. Reduced introgression between melissa and Warner mountains on the Z
Line 292 implies that reduced occurrence of trees clustering melissa and the Warner lineage on the Z is indicative of reduced introgression. In order to account for the confounding effect of Ne on lineage sorting, this needs to be compared to the abundance of other types of non-species trees, in particular the tree grouping Warner with idas. Alternatively the ABBA-BABA test and related f estimators control for this confounding effect.

6. Effect of chromosome length?
Visually, the phylogenetic results appear to agree with the finding of Martin et al. (2019 PLOS Biol) that introgression is generally reduced on longer chromosomes. Can this be tested? As noted above, the distinction between differences in rates of lineage sorting and differences caused by reduced introgression is a key consideration here, and discussed at length in the Martin et al study.

Minor comments

7. In line 109, I think it is worth noting that the elevated LD in DBS is positive or coupling LD, and therefore informative about ancestry.

8. An additional statement in the results addressing directly why two different methods (genomic clines for DBS and ancestry tract analysis for JH) were used, would be useful.

9. Can distinct symbols be used to distinguish the different JH populations in the map and PCA (either in Figure 2 or a supplementary figure)? It is impossible to fully relate the PCA to the map, and also to Figures 2C and S1 without this information.

10. The argument that autocorrelation in window-based phylogenies is more consistent with introgression than ILS (line 286) needs to be expanded a bit for the non-expert reader.

11. Please use the same y-axis scale for Figures 6F, G and H.

12. Trees 1:15 in the phylogenetic analysis should all shown somewhere.

13. I recommend adding further discussion of the implications of these findings for the relative importance of extrinsic and intrinsic barriers in speciation.

Simon Martin

Reviewer #3 (Remarks to the Author):

Chaturvedi et al. reported genomic regions that are likely involved in reproductive isolation ("barrier loci") and selection by using a newly discovered hybrid zone and two ancient hybrid populations of Lycaeides butterflies. Using a contemporary hybrid zone and comparing it with ancient hybrid population/species is very unique and provides new insights into how same genomic regions can be involved in reproductive isolation. The Helianthus sunflower is one of the pioneering systems to investigate such a pattern, but unfortunately, reproductive isolation between the parental species is so strong that it is not possible to investigate the pattern reported in this study.

I do not have much to suggest to further improve the manuscript, but one thing that I am missing is a relationship between the putative barrier loci and chromosomal rearrangements. For instance,

inversions have been known to be associated with barrier loci by suppressing recombination. Dovetail recently released “Omni-C”, which may provide chromosome-level assemblies for whole genome phylogenomics analysis (Line 602) to investigate structural differences between species/populations. Since structural reorganization of genomes appears to be common in butterflies (https://advances.sciencemag.org/content/5/6/eaau3648) and many other species, it is tempting to speculate such a scenario in the Lycaeides system. Perhaps 1-2 lines of discussion about this may be worthwhile.

Specific comments:
Line 110: Figure S4 uses all markers (ie., not only AIM).

Line 197: Figure 5D -> 5F. Also please check the values (35.42 in 1% stringency, which looks like ~40% in Figure 5F)

Line 606-: I could not find summary statistics about “high-coverage”. Please state DP/variant or genome-wide read depth.

Figure S5: Mean minor allele frequency of 1164 AIM?

Response to Reviewer #1:

The paper addresses an important and interesting question, comparing an older hybrid zone with contemporary hybridization. Overall, I think this is a great study that will be broadly interesting, the analyses are thorough, and the paper is very well written. I think the authors did an excellent job of distilling very complex analyses into a concise manuscript. I am very excited about this paper and I enjoyed reading it, but I do have concerns about how some of the results are interpreted.

We are happy the reviewer found the paper interesting and exciting. We have carefully revised the paper to address the reviewer's concerns (see responses below).

The introduction and discussion only considered selection as a mechanism for consistent genomic patterns between ancient and recent hybrids. I think ignoring the recent literature on the role of recombination, gene density, and other structural features on patterns of divergence and shared ancestry is major omission. Structural features of the genome can also lead to shared ancestry tracks, for instance introgressed segments can persist in regions of the genome where there is low recombination. The authors do allude to this on Lines 298 and 299, but I think this is relevant to the discussion about the Jackson Hole/Dubois populations as well.

We agree that recombination rate variation, and especially an interaction between recombination rate variation and selection, can affect patterns of introgression and ancestry in hybrids, and that this can generate associations between recombination rate and introgression (it is the interaction that is critical in the context of our analyses; in the complete absence of selection recombination rate variation will affect the size but not frequency of ancestry blocks, and it is the latter that we consider). We didn't mean to imply that this wasn't the case, and we have corrected this omission in the revised manuscript. First, we now introduce this role for recombination rate variation in the Introduction (rather than saving it for the Discussion) (L26-43). Second, we now test for an association between the degree of introgression and chromosome size (which should be inversely proportional to the average amount of recombination per base pair on a chromosome) in each of the hybrid lineages/zones (as suggested by reviewer 2) (see results on L168-177, 223-227, 356-361, and 366-368). Results from these new analyses suggest some role for variation in recombination rate (at the chromosome scale) in shaping patterns of introgression in *Lycaeides*, although often this pattern is (at least partially) dependent on inclusion of the Z chromosome (e.g., L171-172). At present, we do not have reliable data on fine-scale recombination rates that would allow us to consider the effects of recombination rate at finer scales (which we note in the revised manuscript) (L429-432). We have also expanded our discussion of this in the Discussion (L422-435).

I don't think "ancient" is the right word for the Jackson Hole hybrids. Generally, the term ancient hybridization is used for cases where hybridization is found between allopatric taxa or taxa where one lineage is extinct. What is the evidence that there is no recent hybridization in Jackson Hole

or that the Jackson Hole hybrid populations have progressed towards genome stabilization? I agree that the analyses here suggest the Dubois population is more recent admixture than other Jackson sampling sites and I think the comparisons of these populations is fundamentally interesting. But to me, admixture in the Jackson Hole populations is more consistent with adjacent/intermixed populations that experience periods of contact and backcrossing in a patchy environment (Gompert et al. 2010), rather than an ancient stabilized hybrid lineage.

We respect the reviewer's point of view on this, but we think "ancient" is appropriate when describing the Jackson Hole populations and have revised the text to make our reasoning more clear. This includes an explicit statement about how we use ancient to avoid any ambiguity (L69-70); we also now avoid using this word by itself in the abstract when referring to Jackson Hole (i.e., to avoid ambiguity before defining Jackson Hole *Lycaeides* as ancient). Past work suggests that hybridization between *L. idas* and *L. melissa* occurred ∼10,000 years ago, which we think can accurately be described as ancient (while recognizing that definitions of ancient may vary) (L66-70). At present, at least in the geographic region covered by the Jackson Hole populations, non-admixed *L. idas* are not found. Thus, there isn't really an opportunity for on-going gene flow (in this region) between *L. idas* and *L. melissa*. Instead, Jackson Hole individuals are primarily mating with other Jackson Hole individuals (the Dubois hybrid zone is a clear exception to this). We think that this was unnecessarily confusing because of an error we made in coloring the points on the PCA in the earlier version of the paper that made *L. idas* and Jackson Hole look less distinct than they are (discussed more below). We have corrected this error and hope it clarifies things. Evidence for a lack (or at least rarity) of contemporary hybrids between *L. idas* and *L. melissa* comes from the narrow distribution of admixture proportions/PCA scores within the Jackson Hole populations (compared to Dubois). This is also apparent from past work (e.g., see the histrogram of hybrid indexes in Figure 4 from Gompert et al., 2012). Whenever gene flow from the parental species is low or absent, some progress towards genome stabilization is virtually guaranteed (even if only by genetic drift). Additional evidence for progress towards genome stabilization comes from the ancestry frequency estimates in our current manuscript, which show multiple regions of the genome are fixed or nearly fixed for either *L. idas* or *L. melissa* ancestry. We have endeavored to make this all more clear in the revised manuscript (L103-131, 148-154). We think the patchy environment is (as noted in the reference Gompert et al. 2010) an important part of this pattern as it limits gene flow among populations and thus accelerates progress towards genome stabilization. We agree that there is certainly some gene flow among Jackson Hole populations, but this involves matings between hybrids from nearby populations that have similar genome compositions, not *de novo* hybridization with *L. idas* and *L. melissa*.

As it is currently presented, the discussion and presentation of the admixture results is a bit misleading. Fig 1 shows an idealized version of hybrid indices for an ancient hybrid lineage and

recent hybridization, but only the hybrid indices of Dubois population (recent hybridization) are presented in the main text (e.g. Fig 3A). The distribution of hybrid indices for Jackson Hole is presented in a slightly different format in Fig S1, but does not match the predictions of ancient hybrid lineage presented in Fig 1. Jackson Hole hybrids have hybrid indices close to *L. idas*, with evidence of admixture up to ∼0.20 from *L. melissa*, which I would interpret as backcrossing of rare hybrids into *L. idas*, with little gene flow from *L. idas* allopatric populations. Indeed, in a prior study the ancestry of Jackson Hole populations was so close to *L. idas*, that there was no evidence of admixture using treemix and structure plots generally showed the same patterns between *L. idas* and Jackson Hole, with slightly more *L. melissa* ancestry in Jackson Hole (Gompert et al. 2014, Figs 4, 5, 6). The relative amount of admixture for Jackson Hole populations are not that different from the admixture in what is defined as pure *L. melissa* populations (Fig S1, this manuscript). I think all of this could be made clearer is some version of Fig S1 were included in the main manuscript.

We agree that our presentation was not sufficiently clear and have revised the manuscript to address this issue. We now show the distribution of admixture proportions for Dubois and the Jackson Hole populations in Figure 2 in the main text. We do this both from models that include *L. idas* (and thus where ancestry is polarized/defined relative to *L. idas* and *L. melissa*) and from those that do not (where ancestry is thus polarized relative to Jackson Hole versus *L. melissa*). The corrected PCA also shows more clearly how patterns of structure/ancestry in Jacskon Hole really are different from patterns of structure in *L. melissa* (simple isolation-by-distance with populations sampled over a large geographic area); we now also better emphasize these differences in spatial scale (L118-121). We also discuss patterns of genetic structure in more detail in the main text and in the online supplement (see revised text 103-131 and the new results section in the supplement).

I think the description of the results of the PCA is also a bit misleading. There is not a clear distinction between Jackson Hole hybrids and *L. idas*. There are *L. idas* individuals clustered with Jackson Hole, and vice versa. While there are variants that distinguish these overall populations, the same pattern can be seen in modern humans or any other species that has population structure and isolation by distance. Given the past interpretations of the patchy population structure of these sampling sites, the manuscript should include explicit measures of IBD. Finally, while I agree it is likely that the Dubois population is a product of hybridization between *L. melissa* and Jackson Hole (based on the location of the sites), I don't see how that can be firmly established given that there are both Jackson Hole and *L. idas* individuals clustering with some Dubois individuals in the PCA? Are there additional analyses that support this? Overall, I think the manuscript should be more cautious in interpreting the PCA.

We had incorrectly colored some of the labeled points in the PCA. We apologize for this error, which has been corrected. With this correction, there is a clear distinction between *L. idas* and Jackson Hole *Lycaeides* (see revised Figure 2). We now also include explicit analyses of isolation-by-distance. We show that there is indeed

isolation-by-distance within each taxon (as expected), but that there is also support for increased genetic distances beyond those predicted from simple isolation-by-distance between nominal taxa (see new Fig. S4 and the new results section in the online supplement). This is further supported by new analyses that estimate effective migration rates (see the new results section in the on-line supplement and Fig. S3). We now also include new analyses that show the Dubois population includes individuals that can be classified as Jackson Hole *Lycaeides* and *L. melissa*, but not individuals that can be classified as *L. idas* (L126-128 and Figure S13). Note as well that despite extensive searching for >15 years, we have never encountered "pure" *L. idas* anywhere near the Dubois hybrid zone. This of course can't completely rule out the persistence of an isolated pure population somewhere, but at this point it doesn't seem likely.

All that being said, I think this is a matter of presentation and I don't think that being more cautious in the interpretations would decrease the impact or the appeal of the manuscript. Regardless of how the Jackson Hole hybrids are described, it is still a very interesting and exciting study to compare ancestry in these hybrids relative to new Dubois population.

We have tried to carefully clarify and revise as suggested, and we think that these shifts in presentation have truly improved the paper.

Minor comments:

I think the patterns of consistently restricted introgression on the Z chromosome is indeed interesting, though it isn't particularly exceptional (Line 300). The discussion of factors underlying restricted Z introgression should also mention linkage (modeled in Muirhead and Presgraves, The American Naturalist, 2016).

Our use of the word 'exceptional' here was not meant to indicate that the pattern was exceptional relative to expectations from the literature, but rather relative to the rest of the genome. We have revised the text to make this more clear. We also now make it clear that various factors, including linkage to selected loci, could affect the patterns we observe on the Z chromosome, but also note that in butterflies the Z does not experience reduced recombination relative to autosomes as recombination only occurs in males, which are the homogametic sex (ZZ) (in other words, in females, which are ZW, recombination does not occur between the sex chromosomes, but also does not occur between autosomes) (L408-414). Thanks for this suggestion.

Line 52: "genome collisions" is a bit much (also Line 86, 314).

We have (reluctantly) changed "genome collisions" to "genomic outcomes of hybridization" throughout the paper.

Line 78: citation?

We have added citations regarding the hybrid nature and origin of the Jackson Hole

populations (L66-80).

Line 102: The cluster for Jackson Hole is not particularly tight, and if other populations of Lycaeides were included in the PCA, then Jackson Hole populations cluster with *L. idas*, as presented in Gompert et al. 2014 (Fig 1B).

There was an error in the color coding in this figure and one of the JH sites was swapped with one of the *L. idas* sites (sorry we didn't catch this previously). We have now carefully ensured that our classification and color schemes reflect Gompert et al. 2014 throughout this manuscript. With this correction, Jackson Hole now forms a very tight cluster (with the possible exception of BIC, which is offset some on PC1). Adding additional more divergent species and populations does indeed affect what variation is accounted for on the first PCs and how much populations are compressed or spaced out in PC space. But that doesn't alter the (modest but non-trivial) genetic differentiation between the Jackson Hole and *L. idas* populations.

Fig 1C: Maybe indicate that this is the hypothesized relationship among these populations?

We have modified the figure legend as suggested.

Fig 2: The populations on the map could be related to the PCA through either a gradient of colors or shapes. The shapes/colors could also correspond to the PC scores in Fig 2C. Color blind readers can't distinguish this color palette, and especially in this figure, would miss critical information. The colors should be changed.

We have revised this figure as suggested. We now use shapes to denote different nominal taxa and color gradients to denote populations in the map and PCA. We also now use a color-blind friendly palette. We think these changes do indeed make the figure more informative.

Fig 5 is an excellent summary of results, but it could be easier to read if it were a bit bigger, especially the text?

We have increased the size of the figure and text as suggested.

Fig S2: If this is mean LD shouldn't there be standard deviation or error estimates? Maybe I am misunderstanding how mean LD is calculated.

We have added bars denoting standard errors to the barplots as suggested (note Figure S2 is now S5).

Line 239-242: It is exciting to see a cold tolerance/diapause gene in the list of candidates, especially given the difference in diapause between these two species.

We agree that this is an interesting results and one that we hope to follow-up on in the future.

Line 306-310: It could be said that any study of speciation links macro and microevolutionary processes (in fact, that is how I define the study of speciation), and to be fair many studies make those links much more explicitly than this study, and in a comparative phylogenetic framework. I think this is overselling.

We agree that our statement was too strong, in particular we do not wish to imply that other studies have not linked micro and macroevolutionary processes. We do however think that our results provide a different take on this link by looking at early and late stages of evolution in a hybrid zone/admixed population (this temporal component is not necessarily present in many purported studies of speciation, though it is of course present in some). Thus, we have revised the statement to make it clear that this is but one of various ways such a link can be made (L374-379).

Line 410: typo? "to gene model"

We corrected the typo; this now reads "to gene models". Thanks for catching this.

Line 414: 'partial genome sequences' sounds like whole genome data. It might be clearer to identify these data as GBS. (also on Line 79)

Thanks, we see how this could be confusing and have changed it to genotyping-by-sequencing as suggested (GBS) (L88).

Response to Reviewer 2:

Chaturvedi et al. studied the genomic composition of hybrid-origin lineages of Lycaeides butterflies using a large dataset and a newly-assembled genome. Hybridization provides an exceptional opportunity to study the impacts of selection on the genome and its role in the origin of species. The study system and dataset used here are well suited to address these questions. The main finding of this study is that genomic regions showing extreme ancestry of one or the other parental species in an older (Jackson Hole) hybrid lineage show significant overlap with those showing non-neutral patterns of ancestry (either excess introgression or barrier-like patterns) in a younger (Dubois) hybrid lineage. This highlights an important role for selection in speciation.

Overall I think this is a well-conducted study and a well written paper with results of broad interest. Most of my concerns are about providing more information and interpretation to improve clarity.

Thanks for the positive thoughts on the paper. We have endeavored to address the issues related to interpretation and clarity (see below for details).

1. Developing expectations. The major obstacle for me in getting my head around this study is the fact that the two main focal lineages, Jackson Hole (JH) and Dubois (DBS), are not independent realizations of distinct hybridization events. Rather, the former lineage is thought to be a progenitor of the latter (line 77). While the non-independence is not a fundamental problem, it does alter our expectations. For example in Figure 1, we see how the genomes of ancient hybrids might have

largely stabilized, whereas those of contemporary hybrids can be variable, but if we place this example in the context of the present study, and assume that blue represents *L. melissa* ancestry and gray represents *L. idas* ancestry, then the combination of ancestral and contemporary hybrids shown in Figure 1 would not be possible in the case of JH and DBS: the contemporary hybrids have idas ancestry in parts of their genomes where neither of their parental lineages (JH and melissa) could have contributed it. My point here is not about this schematic figure per se, but that the nuances of this situation need to be better laid out for the reader in the text early on. For example, in parts of the genome where JH is fixed for melissa ancestry do we even expect to be able to distinguish JH and melissa ancestry in the DBS population? (See my next point).

We agree that the nature of the system adds complexity to our paper and results. We have revised the paper to make the expectations for ancestry more clear (L188-194, Fig. 1 legend). First, it is important to note that ancestry is always defined relative to some source/reference population, it is never something absolute. Thus, when we consider Jackson Hole hybrids, we are parsing *L. idas* versus *L. melissa* ancestry, but when we consider Dubois hybrids we instead delineate Jackson Hole versus *L. melissa*. Thus, the diagrams shown are relevant for contemporary hybrids/Dubois so long as one considers the colored blocks to represent chunks inherited from Jackson Hole versus *L. melissa* (this is clarified in the legend for Figure 1)'. It is of course true that blocks in Jackson Hole fixed (or at high frequency) for *L. melissa* ancestry would be hard to distinguish from *L. melissa* ancestry in Dubois, but such blocks are rare (e.g., <4% of the AIMs), and indeed allele frequency differences for the AIMs between Jackson Hole and *L. melissa* are relatively high (for this system/species complex). We have clarified this thinking, as it is central to the manuscript (L188-194).

I also found the order of the first two results sections a bit odd. It is difficult to interpret the clines in the DBS population without first understanding the composition of JH. For example, reading that only 10.9 and 3.5% of the genome of JH is fixed for ancestry of each parental species did change my view of the whole story (again, perhaps Figure 1 is a bit misleading here). I also thought that before seeing Figure S6 it would be useful to see an equivalent figure showing how JH frequencies compare to those in its parental populations. Unless there is good reason not to, I suggest the authors consider placing the section on JH ancestry first in the results.

As noted above, we agree that the complexity of the situation (i.e., Dubois being the product of hybridization between JH [a hybrid] and *L. melissa*) makes it difficult to parse the Dubois cline analyses without first seeing data on the genome composition of the Jackson Hole populations, and we like the suggestion by the reviewer to alleviate this confusion. Thus, we have reordered the results as suggested and now present the ancestry results in JH before moving on to the cline analysis in Dubois. We have also added the suggested quantile-quantile plots for minor allele frequencies in Jackson Hole vs. *L. idas* and *L. melissa* (we present results for three representative JH populations as showing all pairs would be unwieldy) (see new Figures S10-S12).

Also, note our response above regarding ancestry and Figure 1 above.

2. AIMs for JH. The Ancestry Informative Markers used for bgc in DBS are said to be defined based on frequencies in idas and melissa (line 123), but they have (understandably) been applied using JH as a reference population. Given that JH is itself partly of melissa origin, do these AIMs provide sufficient power to distinguish JH and melissa ancestry in DBS in all parts of the genome? For example if there is less melissa ancestry on the Z than on autosomes in JH, then perhaps there is also more power on the Z to detect JH ancestry in DBS. If so, is the finding of greater JH introgression on Z in DBS still valid?

As noted above, most of the AIMs show sufficient genetic differentiation between *L. melissa* and Jackson Hole to detect differentiation introgression in Dubois, with a mean allele frequency difference of 0.28 and 78% with differences of at least 0.1 (see L190-193). We have added this important information to the revised manuscript. Moreover, we now look explicitly at the association between AIM allele frequency differences for Jackson Hole versus *L. melissa* and cline parameter estimates. We show that estimates of α are unassociated with AIM allele frequency differences. In contrast, AIMs with high β estimates have higher AIM allele frequency differences. With that said, AIM allele frequency differences only explain ~7% of the variation in estimates of β and ~21% of the variation in whether these are credibly greater than 0 (i.e., credible evidence of restricted introgression). Thus, most of the information in the cline parameter estimates is not explained by AIM allele frequency differences. We make this clear in the revised manuscript (L205-210). We also note that the relationship between β and AIM allele frequency differences likely has biological and statistical causes. In other words, reduced introgression in the past contributed to the high AIM allele frequency differences and also makes it easier to detect credible evidence of restricted introgression (L210-213).

My above concern was somewhat alleviated when I later saw that AIMs in regions of excess melissa ancestry in JH also show evidence of excess introgression in DBS, but as noted above, I am puzzled about how it is possible to distinguish melissa and JH ancestry in such regions. I expect this apparent paradox and its potential implications for systematic biases will concern other readers too, so it should be directly addressed in the text.

We agree that this result further shows that even in regions of excess *L. melissa* ancestry in Jackson Hole, the allele frequencies in Jackson still differ from the allele frequencies in the specific *L. melissa* populations near the Dubois hybrid zone. This in part arises because of the lack of fixed differences among populations within and between species and because of population genetic structure within species. We have revised the text (as described in the preceding response) to address and clarify this point.

3. Introgression from JH. Again related to the above - of the three significant overlaps in Figure 6, I

was surprised by the overlap between AIMs showing evidence of introgression into DBS and regions of extreme melissa ancestry in JH. Since DBS received melissa-like variation from both parental species, why should the JH alleles introgress and replace those from melissa?

We were initially a bit surprised by this too. Part of the story (we think) is that *L. melissa* is itself variable, so high *L. melissa* ancestry in Jackson Hole does not imply identical (or nearly identical) allele frequencies to specific, current *L. melissa* populations. This is especially evident when one considers that drift (even within genomic regions fixed or nearly fixed for ancestry) in JH has certainly caused allele frequencies to diverge some from *L. melissa* even in *L. melissa* ancestry segments. Beyond that, we think it is interesting that we see excess introgression of JH ancestry in Dubois for genomic regions with high *L. melissa* or *L. idas* ancestry in JH. This would be expected if such alleles/ancestry segments work well together (i.e., epistasis). In other words, selection in JH might have favored certain ancestry combinations across loci that function well together and then that are favored (in combination) by selection in Dubois. We realize this is speculative and simply offer it as one possible explanation for the observed result. We don't speculate on this in the actual manuscript, as the text is becoming long for the required format and we are trying to only add truly necessary additional text (and while this pattern is strong, our specific explanations are speculative).

4. Overlaps. Is there a reason that AIMs showing evidence of introgression (positive alpha) are compared separately to regions that show either excess idas or melissa ancestry in JH, whereas AIMs showing barrier like patterns (positive beta) are compared with a combined 'extreme' category?

We realize this was probably an unnecessary complication. Now in the main text we have six comparisons, positive α, negative α and positive β, each with high *L. idas* or high *L. melissa* ancestry (we then present all comparisons with extreme ancestry in the supplement). Note that the high β with high *L. idas* comparison is quite similar to high β with extreme ancestry (most extreme ancestry was high *L. idas* ancestry). Thus, this has little effect on our results, but adds clarity to the paper (L688-701).

5. Reduced introgression between melissa and Warner mountains on the Z. Line 292 implies that reduced occurrence of trees clustering melissa and the Warner lineage on the Z is indicative of reduced introgression. In order to account for the confounding effect of Ne on lineage sorting, this needs to be compared to the abundance of other types of non-species trees, in particular the tree grouping Warner with idas. Alternatively the ABBA-BABA test and related f estimators control for this confounding effect.

We agree with the reviewer and now calculate f_d as an estimator of admixture from the phylogenetic data. This new analysis supports our previous statement that there is indeed reduced introgression on the Z chromosome. We have updated the methods and main text to include this new analysis (L362-372 and 786-800).

6. Effect of chromosome length? Visually, the phylogenetic results appear to agree with the finding of Martin et al. (2019 PLOS Biol) that introgression is generally reduced on longer chromosomes. Can this be tested? As noted above, the distinction between differences in rates of lineage sorting and differences caused by reduced introgression is a key consideration here, and discussed at length in the Martin et al study.

We have revised the manuscript to analyze the effect of chromosome length/recombination rate introgression and tree toplogies. We do this for ancestry in Jackson Hole (a measure of introgression), cline parameters in Dubois (also a measure of introgression), and tree topologies and admixture proportions (f_d) in the Sierra Nevada and Warner populations. Details vary across these analyses, but in general we find the expected associations between introgression and chromosome length (as predicted when selection occurs on many genes of small/modest effect scattered across the genome), but the signal is often weakened or in some cases erased when the Z chromosome is excluded (Z is one of the largest chromosomes and the most resistant to introgression). We think this does indeed add a nice additional component to the paper, and helps us make a contribution to the current discussion of the effects of selection and recombination on patterns of introgression (and in a novel way given the various comparisons we can make). See Results L168-177, 223-227, 356-361, and 366-368, related text in the Methods, and Figures S17, S20, S29 and S30 for details.

Minor comments

7. In line 109, I think it is worth noting that the elevated LD in DBS is positive or coupling LD, and therefore informative about ancestry.

We agree and have made this change (L121-123).

8. An additional statement in the results addressing directly why two different methods (genomic clines for DBS and ancestry tract analysis for JH) were used, would be useful.

We have clarified this point in the revised manuscript (L140-145, 182-198, 595-601 and 631-637).

9. Can distinct symbols be used to distinguish the different JH populations in the map and PCA (either in Figure 2 or a supplementary figure)? It is impossible to fully relate the PCA to the map, and also to Figures 2C and S1 without this information.

We now use distinct symbols for taxa and colors for populations in the map ad PCA. We agree that this makes both much more clear and easier to cross reference, including making the population structure within taxa more apparent.

10. The argument that autocorrelation in window-based phylogenies is more consistent with introgression than ILS (line 286) needs to be expanded a bit for the non-expert reader.

We have expanded on this in the revised manuscript (L340-343 and Figure S28 legend).

11. Please use the same y-axis scale for Figures 6F, G and H.

This was an oversight and has been corrected in the revised manuscript. Thanks for pointing this out.

12. Trees 1:15 in the phylogenetic analysis should all shown somewhere.

We have added a new supplemental figure that shows all 15 trees, and gives the percentage of inferred trees matching each of the 15 tree topologies (Figure S26).

13. I recommend adding further discussion of the implications of these findings for the relative importance of extrinsic and intrinsic barriers in speciation.

We have expanded on this topic in the discussion of the revised manuscript, while still keeping the discussion brief as per the journal requirements (L402-406).

Response to Reviewer 3:

Chaturvedi et al. reported genomic regions that are likely involved in reproductive isolation ("barrier loci") and selection by using a newly discovered hybrid zone and two ancient hybrid populations of Lycaeides butterflies. Using a contemporary hybrid zone and comparing it with ancient hybrid population/species is very unique and provides new insights into how same genomic regions can be involved in reproductive isolation. The Helianthus sunflower is one of the pioneering systems to investigate such a pattern, but unfortunately, reproductive isolation between the parental species is so strong that it is not possible to investigate the pattern reported in this study.

We agree that this study nicely builds on the impressive work that has been done in *Helianthus* and we are happy to hear that the reviewer found the work unique and novel.

I do not have much to suggest to further improve the manuscript, but one thing that I am missing is a relationship between the putative barrier loci and chromosomal rearrangements. For instance, inversions have been known to be associated with barrier loci by suppressing recombination. Dovetail recently released "Omni-C", which may provide chromosome-level assemblies for whole genome phylogenomics analysis (Line 602) to investigate structural differences between species/populations. Since structural reorganization of genomes appears to be common in butterflies (https://advances.sciencemag.org/content/5/6/eaau3648) and many other species, it is tempting to speculate such a scenario in the Lycaeides system. Perhaps 1-2 lines of discussion about this may be worthwhile.

We agree that the possibility that barrier loci are associated with structural variants such as chromosome rearrangements in quite interesting, and it is something we hope to follow up on in the near future. We now discuss this possibility in the revised manuscript, and do so specifically in the context of associations between chromosome

size (here a proxy for recombination) and introgression (L421-433).

Specific comments: Line 110: Figure S4 uses all markers (ie., not only AIM).

That is correct, here (now Figure S7) we show the allele frequency distribution across all 39,139 SNPs and now make this clear in the figure legend. Means for the AIMs are shown in Figure S8

Line 197: Figure 5D -> 5F. Also please check the values (35.42 in 1% stringency, which looks like ~40% in Figure 5F)

We have done this and ensured that all numbers are consistent.

Line 606-: I could not find summary statistics about "high-coverage". Please state DP/variant or genome-wide read depth.

We have added this information and now avoid the ambiguous term 'high-coverage' (L748).

Figure S5: Mean minor allele frequency of 1164 AIM?

That is correct, we now note in the figure legend that this is the mean across the 1164 AIMs. Note that this is now figure S8 and that we have also added standard error bars to the estimates.

REVIEWERS' COMMENTS:

Reviewer #1 (Remarks to the Author):

The authors have addressed all of my comments. I am glad I could help catch an error in the site labeling on the PCA. That error was what I found most confusing about the manuscript and the interpterion of the JH ancestry (and why I asked for the IBD analyses). The manuscript is definitely clearer, and authors did an excellent job of answering my questions. In my opinion, the terms 'hybrid lineages' and 'hybrid populations' suggest they are the outcome of hybrid speciation. But I would interpret JH as isolated L. idas populations that have experienced introgression in the past (rather than hybrid lineages). Regardless, these terms are not a sticking point for me and I appreciate the authors' perspective.

When reading the IBD section of the supplement, I noticed two typos:
First paragraph: "barrier top gene flow" should be "barrier to gene flow".
First paragraph, 2nd page: "distance along" should be "distance alone"

Reviewer #2 (Remarks to the Author):

The authors have done an admirable job in attending to my concerns. This involved adding several additional analyses, which I think have improved this already impressive paper. I have no further concerns, except for the tiniest of corrections: please check the labels on figures 3C and 6H.

-Simon Martin

Reviewer #3 (Remarks to the Author):

I have no further comments.

Response to Reviewer #1:

The authors have addressed all of my comments. I am glad I could help catch an error in the site labeling on the PCA. That error was what I found most confusing about the manuscript and the interpretation of the JH ancestry (and why I asked for the IBD analyses). The manuscript is definitely clearer, and authors did an excellent job of answering my questions. In my opinion, the terms 'hybrid lineages' and 'hybrid populations' suggest they are the outcome of hybrid speciation. But I would interpret JH as isolated *L. idas* populations that have experienced introgression in the past (rather than hybrid lineages). Regardless, these terms are not a sticking point for me and I appreciate the authors' perspective.

We also see the reviewer's perspective, and agree the our different perspectives are not problematic as terms are used in a clear and consistent manner in the paper.

When reading the IBD section of the supplement, I noticed two typos:

First paragraph: "barrier top gene flow" should be "barrier to gene flow".

First paragraph, 2nd page: "distance along" should be "distance alone"

Thanks, we have fixed these typos (corrected text is in the figure legends for Supplemental Figures 3 and 4).

Response to Reviewer #2:

The authors have done an admirable job in attending to my concerns. This involved adding several additional analyses, which I think have improved this already impressive paper. I have no further concerns, except for the tiniest of corrections: please check the labels on figures 3C and 6H.

We are glad the reviewer was pleased with our revisions and have checked (and corrected) the labels on figures 3C and 6H.

Response to Reviewer #3:

I have no further comments.

We are happy that the reviewer was satisfied with our earlier revisions.
